# Real-time and label-free monitoring of nanoparticle cellular uptake using capacitance-based assays

**DOI:** 10.1038/srep33668

**Published:** 2016-09-19

**Authors:** Rimi Lee, Dong hyun Jo, Sang J. Chung, Hee-Kyung Na, Jeong Hun Kim, Tae Geol Lee

**Affiliations:** 1 Center for Nano-Bio Measurement, Korea Research Institute of Standards and Science Daejeon, Republic of Korea; 2 Fight against Angiogenesis-Related Blindness (FARB) Laboratory, Clinical Research Institute, Seoul National University Hospital, Seoul, Republic of Korea; 3 Department of Biomedical Sciences, College of Medicine, Seoul National University, Seoul, Republic of Korea; 4 Department of Chemistry, College of Natural Science, Dongguk University, 26 Pil-dong 3-ga, Jung-gu, Seoul, Republic of Korea; 5 Department of Ophthalmology, College of Medicine, Seoul National University, Seoul, Republic of Korea; 6 Department of Nanoscience, University of Science and Technology, Daejeon, Republic of Korea

## Abstract

Nanoparticles have shown great potential as vehicles for the delivery of drugs, nucleic acids, and therapeutic proteins; an efficient, high-throughput screening method to analyze nanoparticle interaction with the cytomembrane would substantially improve the efficiency and accuracy of the delivery. Here, we developed a capacitance sensor array that monitored the capacitance values of nanoparticle-treated cells in a real-time manner, without the need for labeling. Upon cellular uptake of the nanoparticles, a capacitance peak was observed at a low frequency (e.g., 100 Hz) as a function of time based on zeta potential changes. In the high frequency region (e.g., 15–20 kHz), the rate of decreasing capacitance slowed as a function of time compared to the cell growth control group, due to increased cytoplasm resistance and decreased membrane capacitance and resistance. The information provided by our capacitance sensor array will be a powerful tool for scientists designing nanoparticles for specific purposes.

Recent years have been witness to a sharp rise in the use of nanomaterials in various scientific fields such as medicine, biomaterial science, and cell and tumor biology^1^,^2^. This has led to the increased development and synthesis of novel nanomaterials. Of these, a series of nano-sized materials such as nanoparticles (NPs), micelles, liposomes and polymeric-drug conjugates has been developed in laboratories and has shown great potential as vehicles for the delivery of drugs, nucleic acids, and therapeutic proteins, among others. Some of these nano-sized materials have entered preclinical studies, while others have already seen success in the pharmaceutical market due to their superior clinical performance over traditional drugs^3^.

In general, *in vitro* cell experiments are used to approximate the effects of NPs on the biological system and in particular to examine the cellular uptake of the NPs. Reports of these experiments have detailed a variety of techniques and reagents based on the use of fluorescent probes^4^,^5^,^6^, electron microscopy^7^,^8^, biochemical assays^9^,^10^ and electrochemical assays^11^,^12^. Unfortunately, the success of most of these methods has been limited to particles with specific properties; in fact, these methods are generally invasive and time consuming, requiring a large number of cells and potentially subject to measurement-related interference. Recently, several groups have reported^13^,^14^ on real-time monitoring of cellular nanoparticle uptake using electric cell-substrate impedance sensors (RT-CES) that measure the alternating current (ac) impedance between the small sensing electrode and the large counter electrode while the cells are cultured on the gold sensing electrode. In the case of RT-CES, the cells attach and spread out on the surface of the sensing electrode, thus blocking the current and causing the electrode impedance to be affected by the shape, adhesion, or mobility of the adherent cells. In all of these impedance measurement methods, cellular uptake of nanoparticles is not observed. Rather, cell adhesion change induced by the cytotoxicity of the nanoparticles is observed.

Here, we present an alternative, label-free approach to detect the cellular uptake of NPs by using capacitance-based sensors to measure the changes in cellular capacitance values in real time. A conceptual schematic diagram is shown in Fig. 1. Our capacitance sensors measure the changes in the dielectric constant (ε), where ε is directly proportional to the capacitance (*C*) via the relation *C* = ε*A/d*, where *A* is the electrode area and *d* is the distance between the two electrodes. In our method, the dielectric constant (ε) noticeably decreases with increasing frequency because while at low frequency the ion current passes between the cells through the cell-cell interactions, at high frequency the ion current penetrates the cell membrane and passes both between and through the cells. Therefore, ε depends on both the composition and volume of the cytoplasm^15^,^16^,^17^. Hence, by measuring capacitance as a function of frequency, it is possible to analyze the properties of both the cell membrane and the cell cytoplasm.

In this study, we monitored the nanoparticles’ cellular uptake with a capacitance sensor array. The presence of NPs in the cytoplasm led to increased cytoplasm resistance and gradually decreasing high frequency slope values. At low frequencies, however, the NPs were undetectable. Our experimental data were in good agreement with the theoretically fitted data obtained by using an equivalent electric circuit and fluorescent images. To our knowledge, this is the first report to monitor cellular uptake of NPs without the use of any labeling.

## Capacitance Measurements of Nanoparticle Cellular Uptake

After seeding 50,000 human umbilical vein endothelial cells (HUVECs) per well, we measured the capacitance at 100 Hz as a function of time while the HUVECs were maintained under normal culture conditions. Concurrently, the same measurements were taken for the HUVECs treated with 10^9^, 10^8^ and 10^7^ particles of amine-modified (positively charged, 200 nm, Supplementary Fig. 1a) polystyrene NPs (NH_3_^+^-PNPs) per well (NH_3_^+^-PNPs are widely known for their cellular uptake^18^). During the cellular uptake of the NH_3_^+^-PNPs, a sharp capacitance peak appeared 1 hour after the PNPs treatment (dotted grey line) as a result of the zeta potential changes caused by the PNPs binding onto the cells and subsequently being internalized. The height of this capacitance peak climbed as the concentration of the NH_3_^+^-PNPs increased (Fig. 2a and Supplementary Fig. 2a), similar to what we observed in our previous reports on antibody receptor mediated endocytosis^19^,^20^.

Based on the change in the capacitance value (*C*) as a function of frequency (*f*), the relationship *C* ∝ *f*^*−a*^ was observed, with low frequencies (*a* = *α*; 0.1–1 kHz) and high frequencies (*a* = *β*; 15–20 kHz) showing two different exponents. We evaluated the time-dependent changes of the |*α|* and |*β|* values respectively, according to the treatment groups. Although the cellular growth control group showed increasing |*β|* values, treating the cells with NH_3_^+^-PNPs resulted in a decreasing pattern of |*β|* values from 1 hour after the PNPs treatment (dotted grey line). The decrease was proportional to the concentration of the NH_3_^+^-PNPs (Fig. 2c, Supplementary Fig. 2c and Supplementary Table 1), so that treatment with 10^9^ PNPs showed the greatest decrease. In contrast, variations in the |*α*| values were more straightforward: the values increased as a function of incubation time (Fig. 2b, Supplementary Fig. 2b and Supplementary Table 1), regardless of whether the PNPs were treated or not.

To understand this phenomenon, we measured the capacitance as a function of frequency (*f*), with and without the PNPs treatment (Supplementary Fig. 3b,c). Detailed |α| and |*β|* values at the low and high frequency regions are given in Supplementary Table 1. Briefly, the difference in the values of |α| and |*β|* for the cell growth control groups at 24 and 48 hours were positive; however, the difference in the |α| values before and after cellular uptake of the PNPs was positive but negative for the |*β|* values. In other words, at high frequency, the rate of decreasing capacitance slowed after cellular uptake of the PNPs, when compared to the cellular growth control group. Based on these measurements, the changes in composition and volume of the cell cytoplasm and nucleus due to the presence of the PNPs were thought to affect the capacitance values in the high frequency region (Supplementary Fig. 3 and Supplementary Note).

To verify that the capacitance peak shown in Fig. 2a and the decreasing |*β|* values at the high frequency region were indeed caused by the uptake of the NH_3_^+^-PNPs, we pre-incubated the HUVECs with chlorpromazine (1 μM) and cytochalasin D (500 nM) – pharmacological inhibitors that interfere with the uptake pathways^21^ (Fig. 2d–f and Supplementary Fig. 2d–f). Under normal culture conditions, the capacitance increased steadily due to the steady increase in the number and size of the cells. During the cellular uptake of the NH_3_^+^-PNPs, a sharp capacitance peak appeared. However, compared to the groups treated with 10^9^ NH_3_^+^-PNPs, a small capacitance peak was observed for the cells treated with chlorpromazine, and those treated with cytochalasin D showed no capacitance peak at all. Furthermore, we evaluated the time-dependent changes of the |*α|* and |*β|* values at the low and high frequency regions, respectively, according to the inhibitor treatment groups. Variations in the |*α|* values were relatively consistent in their patterns, showing increasing values as a function of incubation time (Fig. 2e, Supplementary Fig. 2e and Supplementary Table 1). Remarkably, however, the cytochalasin D-treated groups showed increasing |*β|* values, while the chlorpromazine pretreated groups showed |*β|* values that began to decrease at ~38 hour, after an ~13 hour delay, compared to the groups that began to decrease at ~25 hour after being treated only with the PNPs (Fig. 2f, Supplementary Fig. 2f and Supplementary Table 1).

Time-lapse optical microscope images were recorded concurrently while capacitance measurements were obtained for the 10^9^ NH_3_^+^-PNPs-treated HUVECs (Fig. 3a). We observed the green fluorescent signal from the NH_3_^+^-PNP, which was bound to the cell membrane for about 25 min, and then internalized into the cell cytoplasm area. These images confirm that capacitance increased while the PNPs were bound to the cell membrane and subsequently decreased as the NH_3_^+^-PNP entered and pinched off inside the cell.

We corroborated this by fitting the experimental data using a theoretical electric circuit^22^ (Supplementary Fig. 3a) to obtain theoretically fitted data, which are represented by the symbols in Supplementary Fig. 3b,c. The fitted capacitance data (symbols) aligned relatively well with the experimental data (curved line) for the control group (Set 1; cell growth 24 and 48 hours) and the NPs-treated group (Set 2; cell growth 24 and 48 hours (24 hours after internalization of 10^9^ particles)). The theoretically fitted parameters and procedures are summarized in Supplementary Table 2, Supplementary Figs 3–6 and Supplementary Note. Our observations and simulations suggest that cellular uptake of NPs can be distinguished by measuring the |*β|* values at the high frequency region, which manifest a clearly decreasing pattern.

We performed an immunochemistry staining experiment by staining CD31 in the cell membrane with Alexa594 (red) after measuring the capacitance values; the internalization of the NH_3_^+^-PNPs (green) was qualitatively assessed using confocal microscopy. As Fig. 3b shows, we found that the number of green fluorescent PNPs in the cellular cytoplasm area reflected the degree to which the |*β|* values decreased in the high frequency region. In the following immunofluorescent staining experiments, we further analyzed the cellular uptake of the NH_3_^+^-PNPs in the HUVECs by simultaneously treating them with LysoTracker Red, a membrane-diffusible fluorescent probe that accumulates in acidic organelles such as endosomes and lysosomes. This acidotropic marker labels the compartments involved in the endocytosis processes. At 6 hours after treating the PNPs, we observed an overlap of the green fluorescence of the PNPs and the red fluorescence of the LysoTracker signals. This overlap appeared yellow in color. On the other hand, when pretreated with the inhibitors, confocal imaging showed little to no colocalization of the green-labeled NPs and LysoTracker Red, which supports the results from our pharmacological inhibitor studies (Fig. 3c).

Our capacitance sensor can be applied not only to NH_3_^+^-PNPs but to other NPs, as well. We tested carboxylate-modified PNPs (COO^−^-PNPs, negatively charged, 100 nm; Supplementary Fig. 1a), and similar results were observed for the HUVECs’ uptake of the carboxylate-modified PNPs. All data are shown in Supplementary Figs 7 and 8. The uptake of these NPs resulted in a capacitance peak as a function of time at 100 Hz, and the presence of the PNPs in the cytoplasm area also caused decreases in the |*β|* values at the high frequency region. These NH_3_^+^-PNPs and COO^−^-PNPs experimental results show that capacitance increases when the NPs are bound on the cell membrane and decreases when the NPs are engulfed by the cell.

## Capacitance Measurements of Nanoparticle Cellular Non-Uptake

To determine whether NPs that are not engulfed by the cell show different time- and frequency-dependent capacitance results, we designed two types of polyethylene glycol (PEG)-lipid NPs. PEG-NPs #1 and #2 were labeled with indocyanine green and fluorescein, respectively, via inclusion into the NPs by hydrophobic interactions. Supplementary Fig. 1b,d show the structure, size and zeta potential of the PEG-NPs. It should be noted that steric hindrance caused by the PEG-NPs reduces cellular uptake^23^. PEGs on the NPs reduced cellular uptake (Fig. 4a; red and blue), so a capacitance peak was not observed for the PEG-NPs #1 (177 nm); however, NH_3_^+^-PNPs (200 nm) produced a distinct capacitance peak at 1 hour after the NPs treatment (dotted grey line).

Because TNF-α increases the permeability of the HUVEC monolayer^24^ we measured the capacitance of the HUVECs by treating them first with TNF-α (2 ng/mL) and then later with the PEG-NPs, and found that the cells treated only with the TNF-α showed decreasing capacitance as a function of time starting at 10 hours after the TNF-α was introduced (vertical violet line) (Fig. 4a; solid green line). This decreasing pattern was also evident in the cells treated with both the PEG-NPs and TNF-α (Fig. 4a; dotted olive line). These results are consistent with our previous assertions that decreased capacitance is indicative of increased para-cellular permeability^25^.

To distinguish between the capacitance patterns of ECs that uptake NPs and those that do not, we evaluated the time-dependent changes in the |*α|* and |*β|* values at the low and high frequency regions, respectively. The PEG-NPs #1 without TNF-α treatment showed increased |*β|* values, similar to the control group, but the NH_3_^+^-PNPs that were internalized by the cell cytoplasm showed decreased |*β|* values. TNF-α alone and TNF-α with PEG-NPs also increased the |*β|* values, similar to the control group, although TNF-α with PEG-NPs increased to a slightly lesser degree (Fig. 4c). For PEG-NPs #1, these results indicate that in the absence of TNF-α, R_cyto_, R_m_ and C_m_ are not affected, as the PEGs reduce the cellular uptake of the NPs, and the intracellular electrical properties remain unchanged. In contrast to the pattern observed for the |*β|* values, variations in the |*α*| values reflected real-time capacitance results: the group that contained TNF-α showed decreased |*α|* values at the low frequency region due to the expanded para-cellular region (Fig. 4b). For comparison, we also tested PEG-NPs #2 (122 nm) and COO^−^-PNPs (100 nm), and the results resembled those of the PEG-NPs #1 and NH_3_^+^-PNPs. Data are shown in Fig. 4d–f. The uptake of the COO^−^-PNPs resulted in a capacitance peak as a function of time at 100 Hz but for PEG-NPs #2, no peaks were observed. The presence of COO^−^-PNPs also caused decreases in the |*β|* values whereas the PEG-NPs did not cause this decrease; treatment with the TNF-α or a combination of the TNF-α and PEG-NPs #2 caused decreases in the capacitance and the |*α|* values, and increases in the |*β|* values.

In confocal microscopic experiments, the green fluorescence of the PEG-NPs #1 and #2 was barely observed in the normal states (i.e. absence of TNF-α) of the HUVEC cell membranes (CD31; red). However, in the presence of TNF-α, PEG-NPs #1 and #2 were internalized through cell-cell junctions, which we were able to observe by the colocalization of the cell membrane (CD31; red) and the green fluorescence of the PEG-NPs #1 and #2 (Supplementary Fig. 9a,b). We also analyzed the green fluorescence of the PEG-NPs and the red fluorescence of CD31 on the cell membrane with a correlation test (Pearson’s coefficient) (Supplementary Fig. 9c). These results suggest that NPs that did not enter the cells caused the |*β|* values to increase in the high frequency region, similar to the control group.

## Capacitance Measurements of Lipofectamine Cellular Uptake

After verifying our capacitance system with well-known nanoparticles, we applied our capacitance sensor to a siRNA-lipofectamine 2000 complex to determine its uptake. Real-time capacitance measurements at 100 Hz for the control groups and lipofectamine 2000 alone showed that capacitance increased steadily, and became nearly constant. The results were different for the cellular uptake of the siRNA-lipofectamine 2000 complex. One hour after this treatment (dotted grey line), there appeared a sharp capacitance peak, similar to what was observed for the NH_3_^+^-PNPs and COO^−^-PNPs. After 4 hours, siRNA downregulated CD44 – a protein that is known to mediate cell adhesion – and inhibited the *in vitro* adhesion of the HUVECs matrix^26^, which caused capacitance to decrease (Fig. 5a and Supplementary Fig. 10a). The gene knockdown efficiency as a result of treatment with the siCD44-lipofectamine 2000 complex (25 nM) was measured by real-time PCR. The results showed up to a 57% decrease in the CD44 gene expression when the cells were treated with the siCD44-lipofectamine 2000 complex (Fig. 5d), which aligned with our capacitance observations. At this point we assumed the uptake of the siCD44-lipofectamine 2000 complex by the cells.

To confirm this, we measured the time-dependent changes of the |*α|* and |*β|* values at the low and high frequency regions, respectively, according to the treatment groups. The siCD44-lipofectamine 2000 complex-treated cells showed decreasing |*α|* values as a function of time (due to decreased cellular adhesion), which was consistent with real-time capacitance results. However, the lipofectamine 2000 alone and the control groups showed increasing |*α|* values as a function of incubation time (Fig. 5b and Supplementary Fig. 10b). The |*β*| values of HUVECs treated with the lipofectamine 2000 alone and those treated with the siCD44-lipofectamine 2000 complex showed a distinctive decreasing pattern starting from 1 hour after treatment, compared to the cellular growth control group, which showed increasing |*β*| values (Fig. 5c and Supplementary Fig. 10c). As with the NH_3_^+^-PNPs and COO^−^-PNPs, the sharp capacitance peak and decreasing |*β*| values of the siCD44-lipofectamine 2000 complex-treated cells indicate the liposomes’ entry into the cells.

In addition, CD44 down-regulation reduced cellular adhesion, which in turn resulted in real-time capacitance and |*α|* values to decrease. As with the other NPs, immunofluorescent staining was performed on CD31 in the cell membrane using Alexa594 after the capacitance values were measured and the cells were fixed. Then, the internalization of the siCD44-lipofectamine 2000 complex-FITC was qualitatively assessed using confocal microscopy; green fluorescent liposomes were observed for the cell cytoplasm area (Fig. 5e). We further analyzed the cellular uptake of the siCD44-lipofectamine 2000 complex-FITC in the HUVECs by simultaneously treating them with LysoTracker. At 6 hours after the liposome treatment, we observed an overlap of the green fluorescence of the siCD44-lipofectamine 2000 complex-FITC and the red fluorescence of the LysoTracker signals (Fig. 5f). Based on these results, we determined that the siCD44-lipofectamine 2000 complex entered the HUVEC cells to successfully deliver the gene. For cancer cells such as HeLa cells, we obtained similar time- and frequency-dependent capacitance values in COO^−^-PNPs- and PEG-NPs-treated HeLa cells as those in COO^−^-PNPs- and PEG-NPs-treated HUVEC cells (Supplementary Fig. 11).

## Conclusions

In this report, we used capacitance sensors to monitor the cellular uptake of different types of NPs in a real-time manner by analyzing the absolute capacitance values and their patterns in frequency-capacitance. Upon uptake of the NPs by the target cells, a distinct capacitance peak appeared and the frequency-capacitance slope values in the high frequency region decreased.

The capacitance sensors used here can be easily integrated into an array chip, so it can be used to simultaneously monitor the various cellular-uptake related phenomena regarding the surface properties of numerous fabricated NPs. Based on this information, we can then pinpoint the specific surface properties that determine whether uptake occurred, which is key to designing and producing NPs for a specific purpose in a systematic manner.

Another exciting feature is that the capacitance sensor array is fabricated on a glass substrate and the cells are seeded between the electrodes, meaning that optical measurements can be taken alongside capacitance measurements in a real-time manner. After successfully recording the capacitance values, immunocytochemistry or other molecular studies involving cells can be carried out by referring to the capacitance measurements. It is important to note that the capacitance values are unaffected by the existence of the NPs, unlike conventional imaging observation methods that are often subject to interference by the fluorescent NPs.

This novel system is a label-free and real-time *in vitro* method for high-throughput screening to identify the cellular uptake of different types of NPs including therapeutic nanomaterials and to monitor their effects on cellular states. We are confident that the findings contained in this report will prompt promising applications of our system, including designing NPs with a specific purpose.

## Methods

### Fabrication of Capacitance Sensors

**Figure 1:**
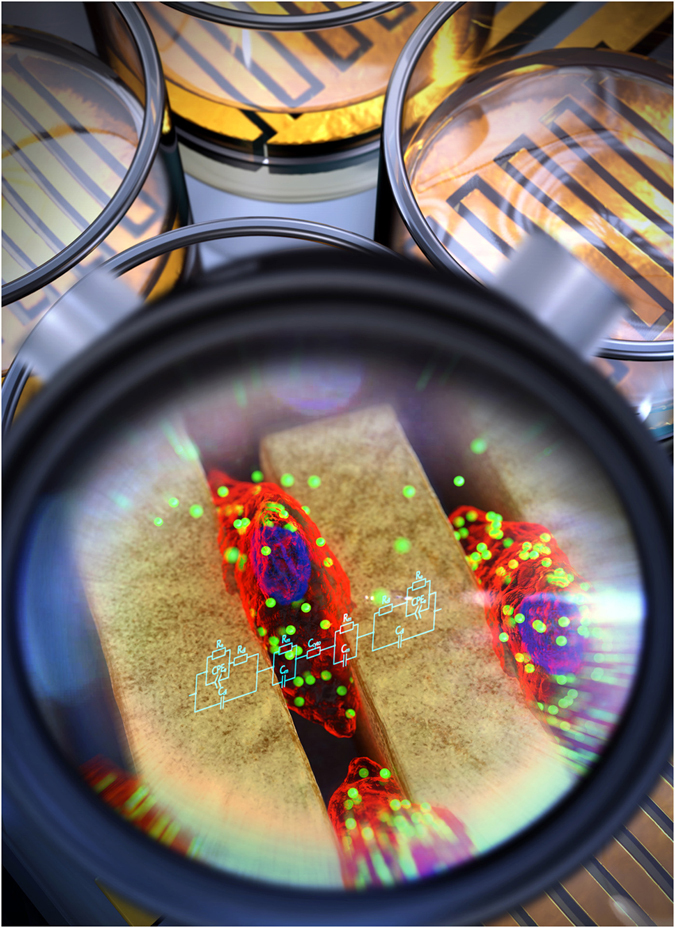
Conceptual schematic diagram of our capacitance sensor. The capacitance sensor array was composed of 16 well sensors with interdigitated Au electrodes on a glass substrate. Acrylic wells were attached to the array. The structure of this sensor allows for optical and capacitance measurements to be taken simultaneously in real-time. Red = cell cytoplasm; blue = cell nucleus; green = NPs; sky blue =  electric circuit of the cell and capacitance sensor.

We fabricated a capacitance sensor array composed of 16 well sensors with interdigitated electrodes on a glass substrate (Fig. 1 and Supplementary Fig. 12). Using photolithography and lift-off techniques, 100 nm-thick Au electrodes were patterned with a gap size of 30 μm. A 50 nm-thick SiO_2_ layer was deposited on top of the Au electrodes to minimize the influence of the cells on the capacitance readings. Then, acrylic wells from Lab-Tek Chamber Slide (Lot no. 10118584) were attached to the array with a curing agent for the cell culture. All of the experiments took place after sterilizing the capacitance sensors in an autoclave.

### Cells and Nanoparticles

Human umbilical vein endothelial cells (HUVECs; ATCC) that contained 2% FBS and VEGF for rapid proliferation (Lonza) were maintained with Endothelial Cell Growth Medium (EGM)-2 BulletKit. The cells were incubated in an atmosphere of 95% air and 5% CO_2_ at 37 °C. To induce alteration permeability, the media were exchanged with EGM containing TNF-α (2 ng/mL) for 24 hours.

We used two types of fluorescently labeled polystyrene NPs (PNPs), two types of polyethylene glycol (PEG)-coated lipid NPs (PEG-NPs) and two types of liposomes. The structure, size and zeta potential of the NPs and liposomes are summarized in Supplementary Fig. 1. The PNPs were purchased from Thermo Fisher Scientific (Waltham, MA). Amine-modified (NH_3_^+^, positively charged, 200 nm with a surface charge of 260 μEq/g, Lot number F8764) and carboxylate-modified (COO^−^, negatively charged, 100 nm diameter with a surface charge of −283.9 μEq/g, Lot number F8803) PNPs were used. The excitation/emission wavelengths for the amine- and carboxylate-modified PNPs were 405/515 nm (yellow green fluorescence).

The structure of our PEG-NPs was HO-PEG(1000)-Glutamic acid (C_10_H_21_)-C_8_H_17_. The surfaces of the particles displayed OH groups from the PEG polymer, and two aliphatic tails made up the inner part of the particle rendering the lipid NPs neutral. The PEG-NPs#1 (174 nm) contained indocyanine green (0.04 mg/mL) within the overall structure of the NPs (1.4 mg/mL) as a result of non-covalent interactions. PEG-NPs#2 (122 nm) contained fluorescein rather than indocyanine green.

Lipofactamine 2000 (420 nm) was purchased from Invitrogen (Carlsbad, California, USA) to act as a liposome-based gene transfection reagent. Small interfering RNA (siRNA) to target CD44 and primer for real-time PCR were purchased in validated sequence from Bioneer (#1028125, Daejeon, Korea). Transfection protocols were followed according to the manufacturer’s instructions. All experiments were carried out in triplicate; the error bars represent the standard deviation.

The number of microspheres per mL of suspension was determined from the following equation:


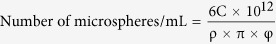


where: C = concentration of suspended beads in g/mL

φ = diameter of microspheres in μm

ρ = density of polymer in g/mL (1.05 for polystyrene)

The structure, size, TEM image and zeta potential of the NPs used in this study are given in Supplementary Fig. 1. The zeta potentials and hydrodynamic sizes of the materials were measured by dynamic light scattering analysis using a Zetasizer Nano ZSP (Malvern instruments, UK). TEM images were acquired by JEM-1011(JEOL, Japan).

### Measurement of Capacitance and Theoretical Simulation

Prior to cell seeding, each sensor was sterilized in an autoclave. HUVECs (50,000 cells per well for a confluent culture) were plated onto each well and incubated for 24 hours in the incubator in an atmosphere of 95% air and 5% CO_2_ at 37 °C. Then, the media was exchanged for one containing NPs. To induce alterations in permeability, the media was changed to one containing TNF-α (2 ng/mL) and the cells were incubated for a further 24 hours, after which the media was again exchanged for one containing NPs. Capacitance was measured after the initial seeding of the HUVECs in the wells using the Precision Impedance Analyzer (cat. no. 4294A, Agilent) with an AC voltage of 10 mV at a frequency range of 0.1–100 kHz. Data were collected every 5 minutes from each sensor with a data acquisition/data logger switch unit (cat. no. 34970A, Agilent) connected to an impedance analyzer. Based on the change in the capacitance value (*C*) as a function of frequency (*f*), the relationship *C* ∝ *f*^*−a*^ was observed, with low frequencies (*a* = *α*; 0.1–1 kHz) and high frequencies (*a* = *β*; 15–20 kHz) showing two different exponents. In the log-log plot (Supplementary Fig. 3b,c), the value of −*a* (−*α* or −*β*) indicates the slope in the relationship log*C* ∝ −*a*log*f*. To understand the changes in the α and *β* values at the low and high frequency regions, respectively, a theoretical simulation was carried out by fitting the measured capacitance data with a theoretical electric circuit using the Zview program (Scribner Associates Inc.).

### Immunocytochemistry

After recording the capacitance values, the media was removed from each well. Then, the cells were fixed with 4% paraformaldehyde for 15 minutes at room temperature (RT). Permeabilization of the cells was performed with 0.25% Triton X-100 in PBS for 15 minutes at RT. To minimize non-specific binding, 3% bovine serum albumin (BSA) was applied to the cells for 10 minutes also at RT. After incubation, the cells were treated with anti-CD31 antibody (PECAM-1, dilution 1:200) in 1% BSA-PBS and incubated overnight at 4 °C. Then, goat-anti-rabbit secondary antibody (Alexa Fluor 594, Invitrogen, dilution 1:500) in 1% BSA-PBS buffer was added and left to incubate in the dark for 1 hour at RT. Nuclear staining was performed with 4′,6-diamidino-2-phenylindole (cat. no. D1306, Invitrogen, dilution 1:1000) for 10 minutes at RT. The cells were observed using a confocal microscope (IX81/FV300; Olympus, Japan) after instillation of the mounting solution.

### Pharmacological Inhibition Study

For our study on pharmacological inhibitors, HUVECs were seeded (50,000 cells per well) in a 16 well sensor plate and then allowed to adhere for 24 hours. Chlorpromazine hydrochloride (Sigma, C8138) and cytochalasin D (Sigma, C2618) were added to the cells at concentrations of 1 μM and 500 nM, respectively. The cells were incubated for 1 hour, after which the NPs were added to the cells (10^10^ PNPs/well) containing the inhibitors for 23 more hours. For live cell imaging, HUVECs were seeded onto custom-made glass bottom dishes (MaTek cultureware, Ashland, MA. USA), and grown to approximately 10–20% confluence (20,000 cells/well). The cells were then exposed to 10^10^ PNPs with and without inhibitors for 6 hours in an incubator consisting of 5% CO_2_ at 37 °C. After the PNPs treatment, the unbound NPs were rinsed off and the treated cells were incubated with 10 μM/mL of LysoTracker Red DND-99 (Invitrogen, Carlsbad, CA) in serum free EGM-2 for 30 minutes at RT and imaged with a confocal microscope (IX81/FV300; Olympus, Japan).

### Cytotoxicity Assay

After measuring the capacitance, the 16 well sensor plates were removed from the impedance analyzer. To measure cell viability, trypan blue exclusion assay was performed by staining the cells with trypan blue solution (0.02% in final concentration) for 5 minutes at RT. Immediately after, the trypan blue-negative viable cells were counted using a Countess Automated Cell Counter (Invitrogen, Carlsbad, CA, US) in accordance with the manufacturer’s instructions (Supplementary Fig. 13).

### Statistical Analysis

**Figure 2:**
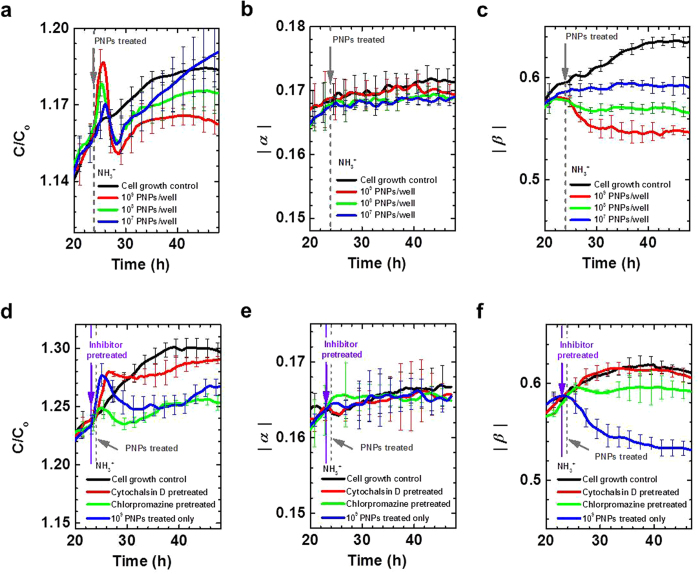
Time- and frequency-dependent capacitance values for amine modified polystyrene nanoparticles. (**a**) Time-dependent normalized capacitance values, *C/C*_*o*_, where *C*_*o*_ is the initial capacitance for HUVEC uptake of different concentrations (10^7^, 10^8^, and 10^9^ PNPs/well) of amine-modified PNPs. The NPs were treated at 23 hour of incubation (gray arrow). After the initial measurement at 100 Hz, the capacitance was measured at various frequencies from 100 Hz to 20 kHz. The capacitance readings were fitted to the relationship *C* ∝ *f*^*−α*^ and *C* ∝ *f*^*−β*^ in the frequency range of 100 Hz to 1 kHz and of 15 kHz to 20 kHz, respectively. (**b,c**) Time-dependent estimates of |α| **(b)** and |β| **(c)** from real-time capacitance measurements using a capacitance sensor array (n = 5). (**d**) Time-dependent normalized capacitance values, *C/C*_*o*_, where *C*_*o*_ is the initial capacitance for HUVECs pretreated with chlorpromazine and cytochalasin D at 23 hour (violet arrow), 1 hour before treatment with NPs (gray arrow). After the initial measurement at 100 Hz, capacitance was measured at various frequencies from 100 Hz to 20 kHz. Time-dependent estimates of α **(e)** and β **(f)** from real-time capacitance measurements using a capacitance sensor array (n = 5). Full data are presented in Supplementary Fig. 2.

**Figure 3:**
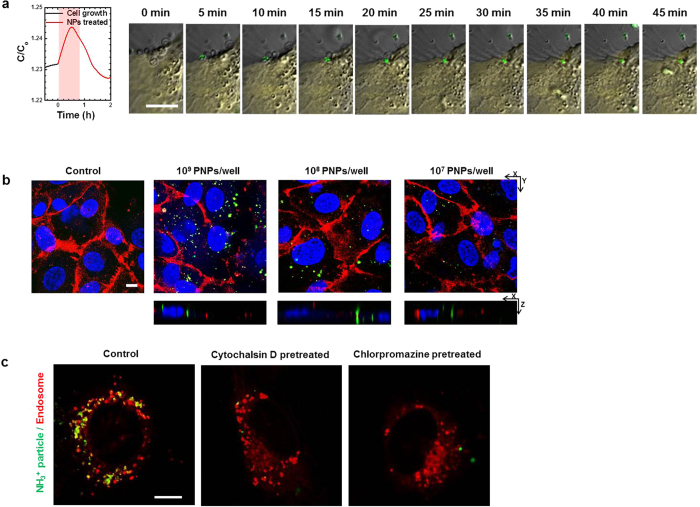
Images of HUVECs treated with amine modified polystyrene nanoparticles. (**a**) Real-time capacitance results and time lapse confocal images of HUVECs during the internalization of the NH_3_^+^-PNPs (green) into the cell membrane (light yellow area). (**b**) Confocal microscopy of amine-modified PNPs uptake by HUVECs. Confocal cross-section and z-stack images of HUVECs show internalization of NH_3_^+^-PNPs 10^9^, 10^8^ and 10^7^ PNPs/well. Scale bars, 10 μm and stack depth, 8 μm. (**c**) HUVECs’ uptake of both NH_3_^+^-PNPs (green) and LysoTracker^®^ Red (left); the same, pretreated with cytochalasin D (middle); and the same, pretreated with chlorpromazine (right). The yellow regions indicate co-localization of NH_3_^+^-PNPs with LysoTracker in the superimposed images. Scale bars, 10 μm.

**Figure 4:**
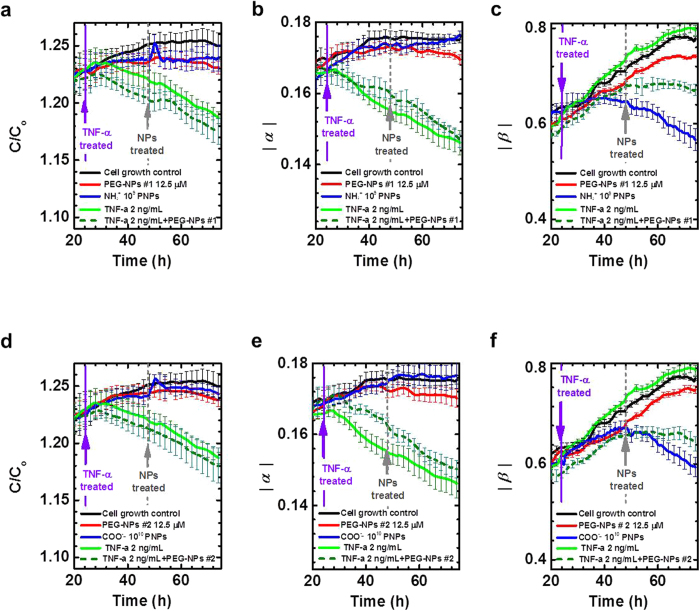
Time- and frequency-dependent capacitance values in PEG-NPs treated cells. Time-dependent normalized capacitance values, *C/C*_*o*_, where *C*_*o*_ is the initial capacitance for HUVECs treated with TNF-α at 24 hours (violet arrow); PEG-NPs#1 and NH_3_^+^-PNPs **(a)**; and PEG-NPs#2 and COO^−^-PNPs **(d)** treated at 48 hours (gray arrow). After the initial measurement at 100 Hz, the capacitance was measured at various frequencies from 100 Hz to 20 kHz. Time-dependent estimates of α **(b,e)** and β **(c,f)** from real-time capacitance measurements using our capacitance sensor array (n = 5).

**Figure 5:**
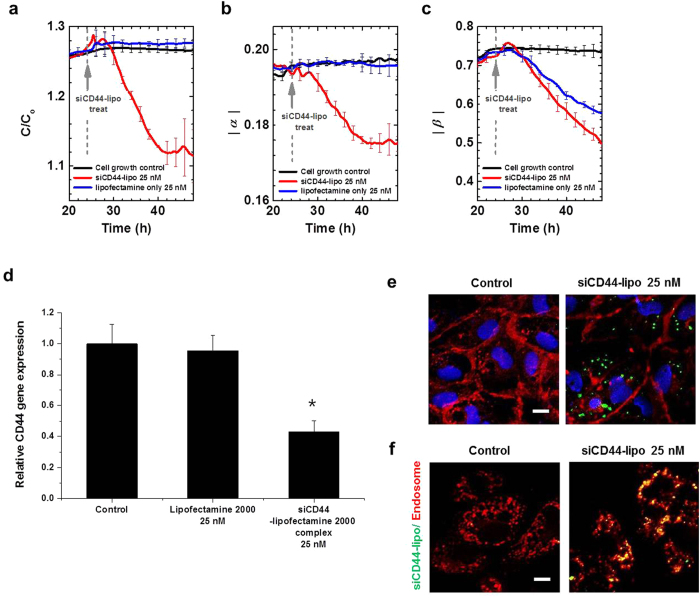
Time- and frequency-dependent capacitance values in siCD44-lipofectamine 2000 treated cells. (**a**) Time-dependent normalized capacitance values for HUVECs treated with lipofectamine 2000 alone and siCD44-lipofectamine 2000 at 24 hour (gray arrow). Time-dependent estimates of α (**b**) and β (**c**) from real-time capacitance measurements using a capacitance sensor array (n = 5). (**d**) The relative gene expression level of CD44 measured by real-time PCR (n = 3). *vs control, *P* < 0.001. (**e**) Confocal cross-section images of HUVECs showing internalization of siCD44-lipofectamine 2000 complex with FITC (green). (**f**) HUVECs uptakes of siCD44-lipofectamine 2000 complex with FITC (green) and then treated with LysoTracker^®^ Red. The yellow regions indicate co-localization of siCD44-lipofectamine 2000 complex with LysoTracker in the superimposed images. Scale bars, 10 μm. Full data are presented in Supplementary Fig. 10.

Data in Figs 2, 4 and 5 are shown in mean values ± standard deviation of at least three independent experiments. The number of samples used in each experiment is noted in each Figure. Significant differences were determined based on the Student’s t-test. Statistical analyses were performed with GraphPad Software. P-values less than 0.001 were considered to be statistically significant.

## Additional Information

**How to cite this article**: Lee, R. *et al*. Real-time and label-free monitoring of nanoparticle cellular uptake using capacitance-based assays. *Sci. Rep.* **6**, 33668; doi: 10.1038/srep33668 (2016).

## Supplementary Material

Supplementary Information
