# Supplementary material for: Real-time and label-free monitoring of nanoparticle cellular uptake using capacitance-based assays
Source: Sci Rep. 2016 Sep 19;6:33668. doi: 10.1038/srep33668 (PMC5027564; doi:10.1038/srep33668)
Supplement: Supplementary Information [file srep33668-s1.pdf]

# Supplementary information

# Real-time and label-free monitoring of nanoparticle cellular uptake using capacitance-based assays

Rimi Lee[1], Dong hyun Jo[2,3], Sang J. Chung[4], Hee-Kyung Na[1], Jeong Hun Kim[2,3,5,*] and Tae Geol Lee[1,6,*]

[1] Center for Nano-Bio Measurement, Korea Research Institute of Standards and Science Daejeon, Republic of Korea

[2] Fight against Angiogenesis-Related Blindness (FARB) Laboratory, Clinical Research Institute, Seoul National University Hospital, Seoul, Republic of Korea

[3] Department of Biomedical Sciences, College of Medicine, Seoul National University, Seoul, Republic of Korea

[4] Department of Chemistry, College of Natural Science, Dongguk University, 26 Pil-dong 3-ga, Jung-gu, Seoul, Republic of Korea

[5] Department of Ophthalmology, College of Medicine, Seoul National University, Seoul, Republic of Korea

[6] Department of Nanoscience, University of Science and Technology, Daejeon, Republic of Korea

*Correspondence authors: tglee@kriss.re.kr (Tae Geol Lee), steph25@snu.ac.kr (Jeong Hun Kim)

## Table of Contents


| | Contents | page |
|---|---|---|
| **Supplementary Figure 1** | Structure, size and zeta-potential of NPs | **3** |
| **Supplementary Figure 2** | Time- and frequency-dependent capacitance values in amine-modified PNPs-treated HUVEC cells | **4** |
| **Supplementary Figure 3** | Frequency-dependent capacitance results for HUVECs, with fitting simulations | **5** |
| **Supplementary Figure 4** | Interfacial polarization in cell suspension | **6** |
| **Supplementary Figure 5** | Frequency-dependent capacitance results for HUVECs, with re-fitting simulations to validate fitting procedures | **7** |
| **Supplementary Figure 6** | Frequency-dependent capacitance results for HUVECs, with fitting simulations for changes in cell-cell or cell-matrix interactions | **8** |
| **Supplementary Figure 7** | Time- and frequency-dependent capacitance values in carboxylate-modified PNPs-treated HUVEC cells | **9** |
| **Supplementary Figure 8** | Images of carboxylate-modified PNPs-treated HUVECS cells | **10** |
| **Supplementary Figure 9** | Images of PEG-NPs-treated HUVECS cells and correlation coefficients | **11** |
| **Supplementary Figure 10** | Time- and frequency-dependent capacitance values in liposome-treated HUVEC cells | **12** |
| **Supplementary Figure 11** | Time- and frequency-dependent capacitance values in carboxylate-modified PNPs- and PEG-NPs-treated HeLa cells | **13** |
| **Supplementary Figure 12** | Schematic diagram of a capacitance sensor array | **14** |
| **Supplementary Figure 13** | Cell viability of NPs and chemicals used in this study | **15** |
| **Supplementary note** | Frequency-dependent capacitance values in cell growth control groups and amine-modified PNPs-treated HUVEC cells with fitting simulations | **16-21** |
| **Supplementary Table 1** | Difference in $\|\alpha\|$ and $\|\beta\|$ values at the low and high frequency regions, respectively, at 24 h and 48 h (NP treatment at 24 h) | **22** |
| **Supplementary Table 2** | Estimated parameters for capacitance-based cellular sensors and percentage of change for each parameter | **23** |
| **References** | | **24** |

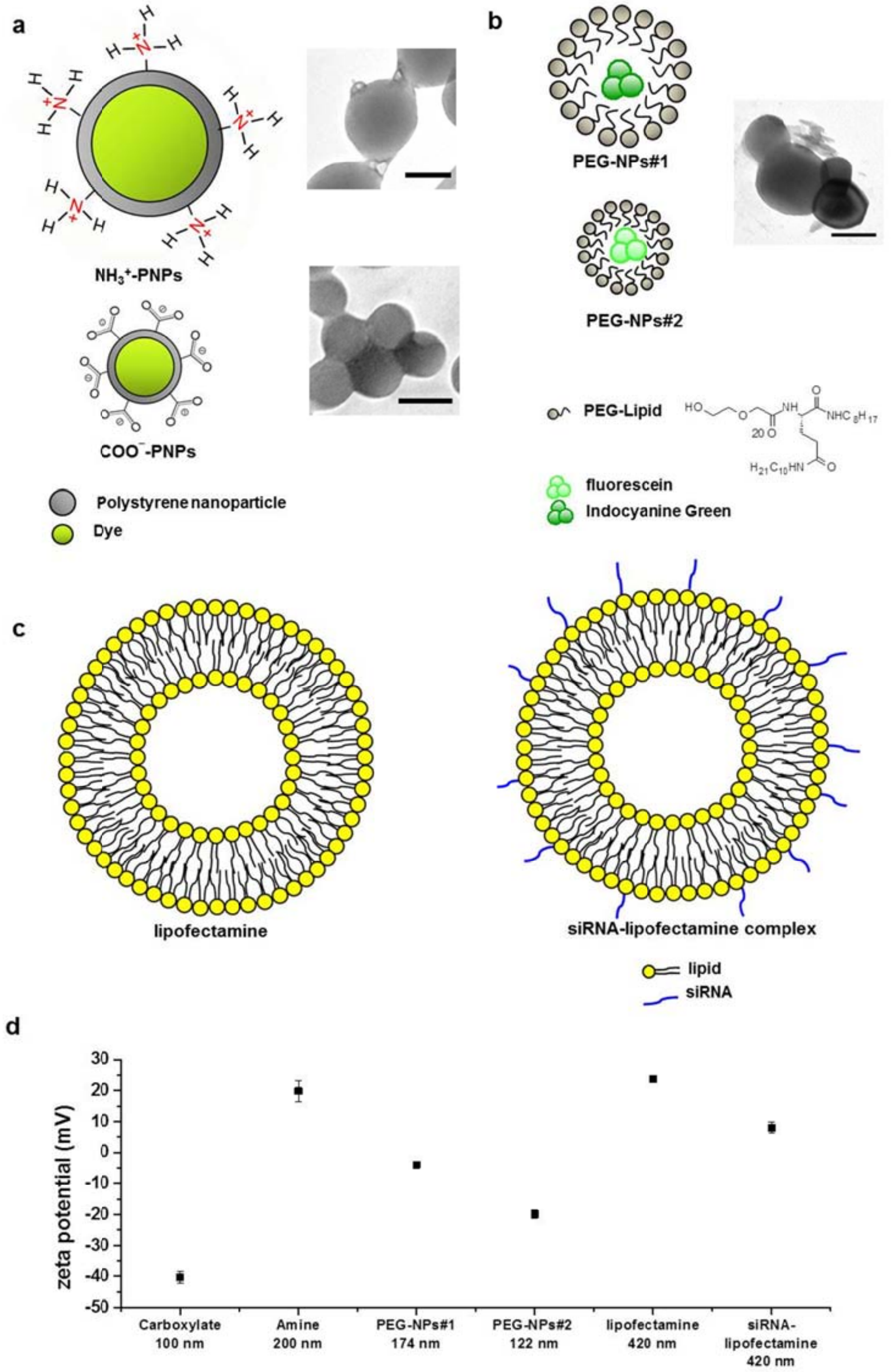


**Supplementary Figure 1.** Structure, size and zeta-potential of NPs. (a) Structure of amine- and carboxylate-modified PNPs and TEM image. Scale bars are 200 nm and 100 nm, respectively. (b) Structure of PEG-NPs and TEM image. Scale bar is 100 nm. (c) Structure of liposome and siCD44-liposome. (d), Size and zeta-potential of NPs used in this study.

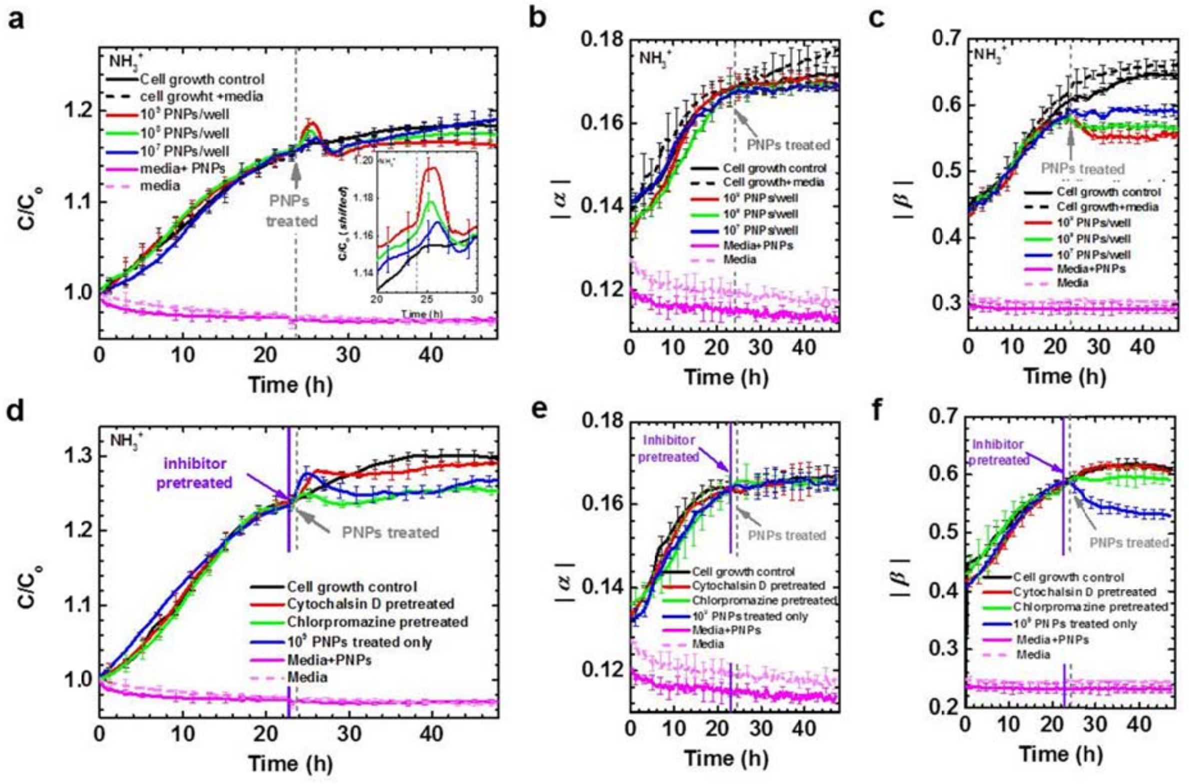


**Supplementary Figure 2.** Time- and frequency-dependent capacitance values in amine-modified PNPs-treated HUVEC cells. (a) Time-dependent normalized capacitance values for HUVECs uptake of different concentrations ($10^7$, $10^8$, and $10^9$ PNPs/well) of amine-modified PNPs. The PNPs were treated at 24 hours of incubation (gray arrow); the inset graph shows capacitance peaks after the PNPs treatment. (The data have been shifted for clarity.) The capacitance readings were fitted to the relationship $C \propto f^{-\alpha}$ and $C \propto f^{-\beta}$ in the frequency range of 100 Hz to 1 kHz and 15 kHz to 20 kHz, respectively. (b, c) Time-dependent estimates of $|\alpha|$ (b) and $|\beta|$ (c) values from real-time capacitance measurements using our capacitance sensor array. (d) Time-dependent normalized capacitance values, $C/C_o$, where $C_o$ is the initial capacitance for HUVECs pretreated with chlorpromazine and cytochalasin D at 23 hour (violet arrow), 1 hour before the amine-modified PNPs treatment (gray arrow). Time-dependent estimates of $|\alpha|$ (e) and $|\beta|$ (f) values from real-time capacitance measurements using a capacitance sensor array (n=5).

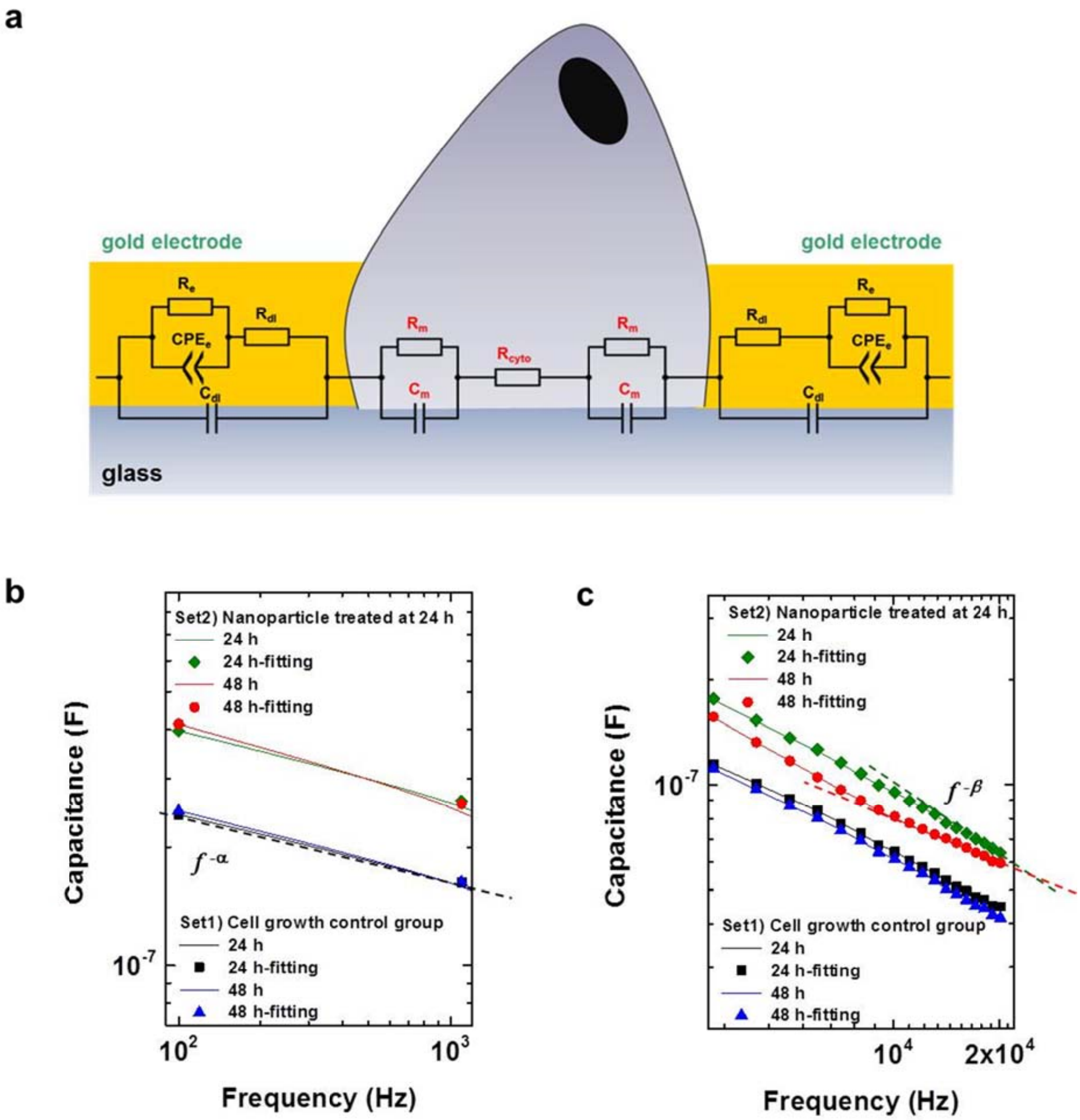


**Supplementary Figure 3.** Frequency-dependent capacitance results for HUVECs, with fitting simulations. (a) Electric circuit of the cell and capacitance sensor. (b, c) Fitted values at low frequencies (b) and high frequencies (c). The black and blue colors represent the cellular growth control groups at 24 and 48 hours, respectively. The green and red colors represent the NPs-treated groups. The solid lines represent the capacitance data, and the symbols represent the fitted values using the electric circuit shown in (a). The calculated fitted values are summarized in **STable 2a and b**.

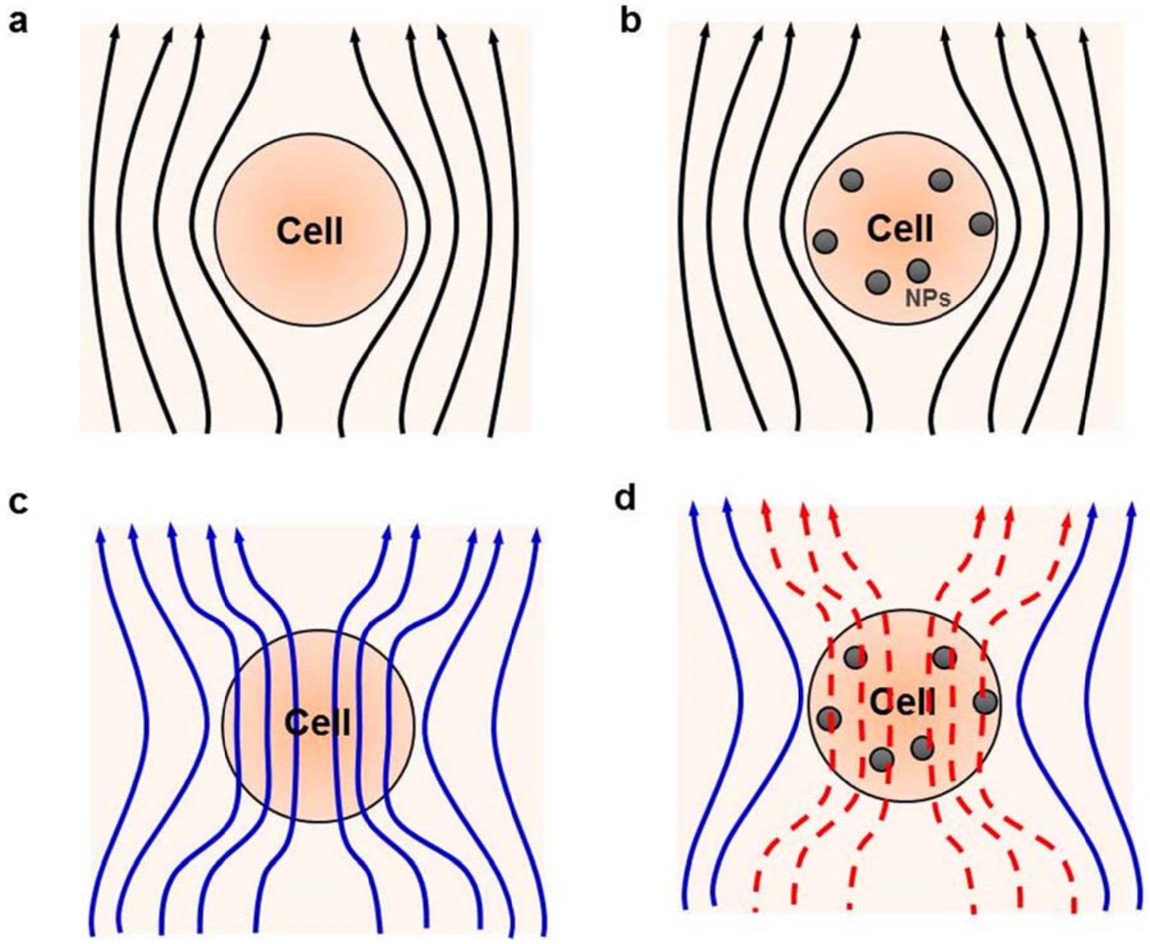


**Supplementary Figure 4.** Interfacial polarization in cell suspension. Interfacial polarization at low frequencies (a) with uptake of NPs (b). Interfacial polarization at high frequencies (c) with uptake of NPs (d). Shown are the lines of the ion currents that flow around the cell after being placed in a uniform AC field.

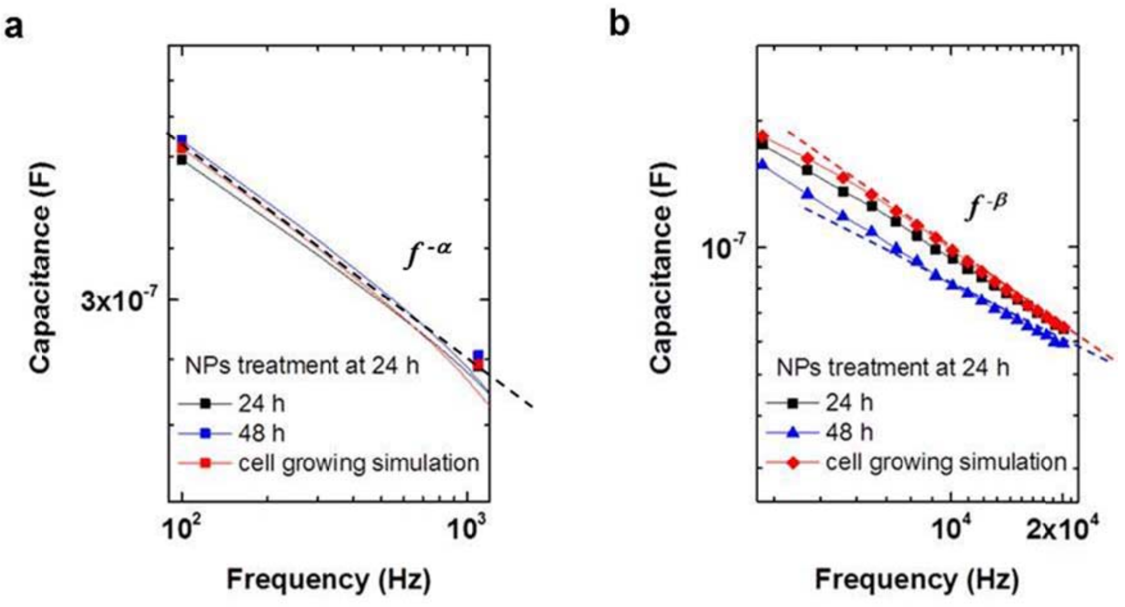


**Supplementary Figure 5.** Frequency-dependent capacitance results for HUVECs, with re-fitting simulations to validate fitting procedures. Fitted values at low frequencies (a) and at high frequencies (b); the black and blue colors represent the cellular growth control group at 24 hours and the NPs-treated group at 48 hours (after NPs treatment at 24 hours), respectively, for Set 2. The red color represents re-fitting simulation of the cellular growth control group at 48 hours. For the re-fitting simulations, the fitted values were obtained by using the electric circuit shown in Supplementary Fig. 3a after fixing the parameter values related to the electrode ($R_{dl}$, $C_{dl}$, $R_e$, CPEe-T and CPEe-P) to those obtained from Set 2 at 24 hours. Only $R_{cyto}$, $R_m$ and $C_m$ were adjusted according to the percentages obtained from Set 1. The theoretically simulated values are summarized in **STable 2b**: Cell growth/Simulation.

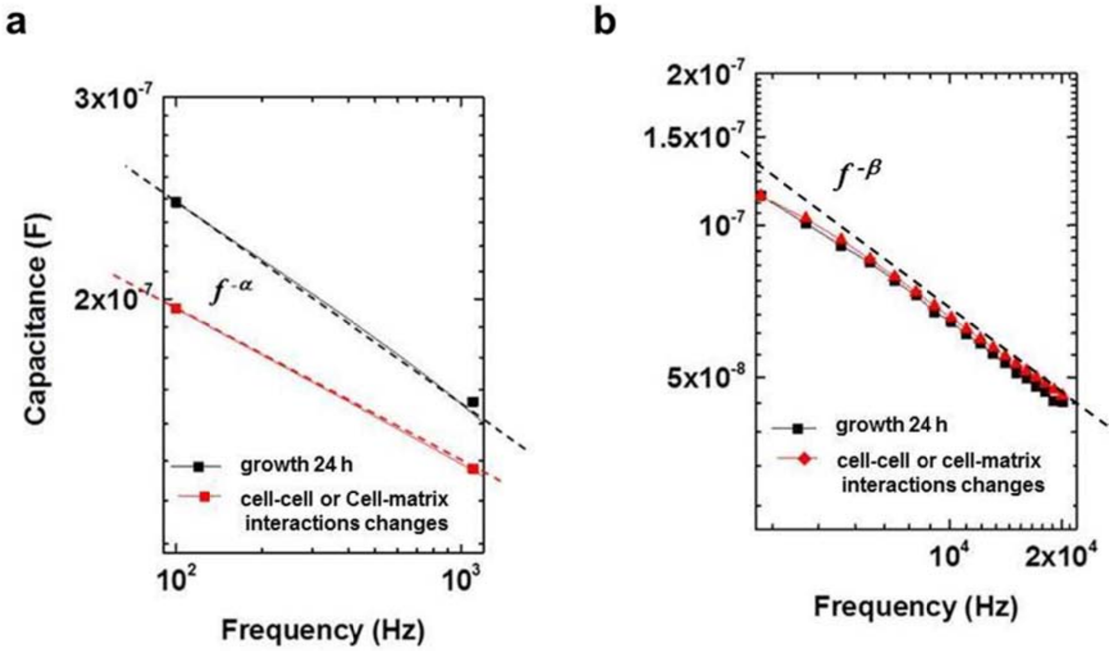


**Supplementary Figure 6.** Frequency-dependent capacitance results for HUVECs, with fitting simulations for changes in cell-cell or cell-matrix interactions. Simulation values at low frequencies (a) and at high frequencies (b); the black color represents the cellular growth control group at 24 hours and the red color represents the simulation of cell-cell or cell-matrix interactions changes. For fitting simulations, the fitted values were obtained by using the electric circuit shown in Supplement Fig. 3a after fixing the parameter values related to resistance ($R_{cyto}$, $R_{m,}$ $R_{dl}$ and $R_e$) to those obtained from Set 1 at 24 hours. Only $C_m$, $C_{dl}$ and $CPE_e$ were adjusted. The theoretically simulated values are summarized in **STable 2a**: Cell-cell or cell-matrix interactions changes/Simulation. The solid lines represent the capacitance measurements data and the symbols show fitted values using the electric circuit shown in **SFigure 3a**.

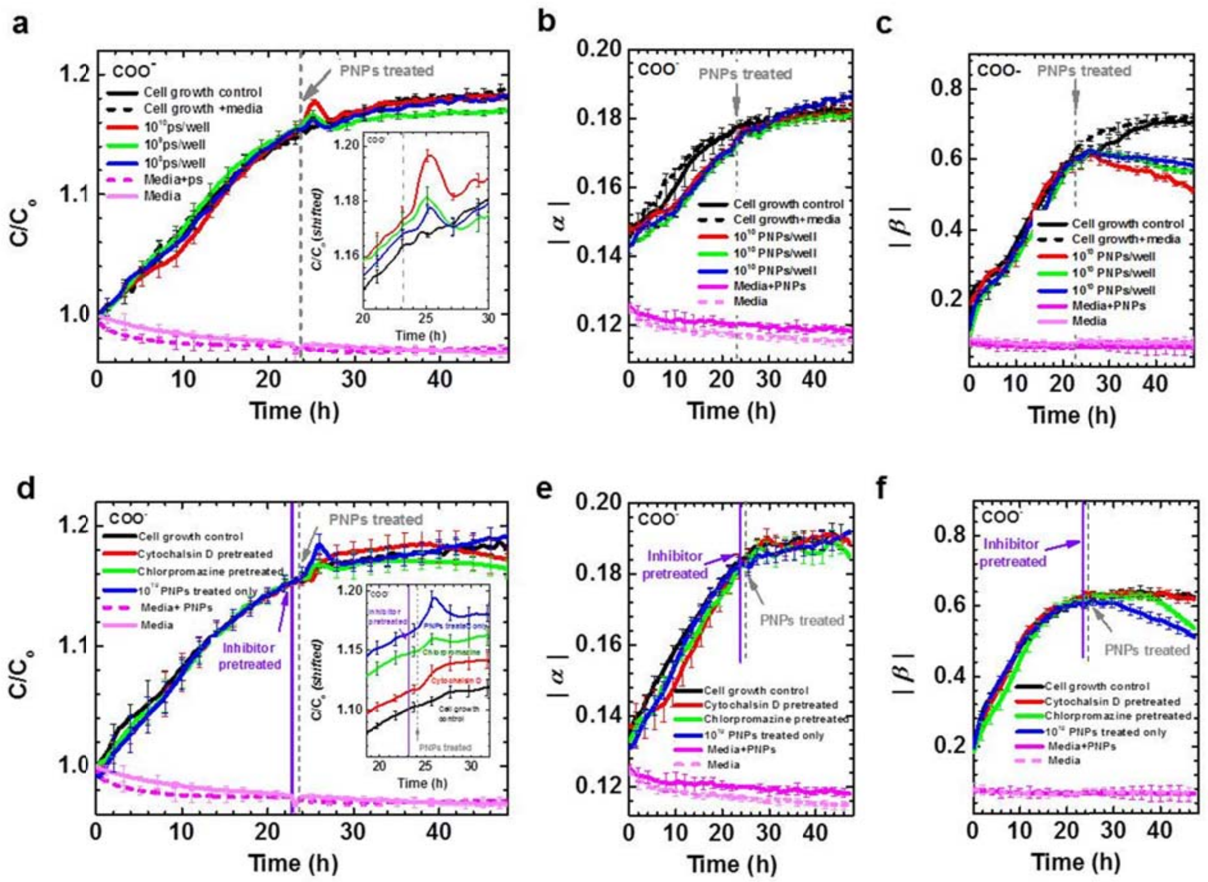


**Supplementary Figure 7.** Time- and frequency-dependent capacitance values in carboxylate-modified PNPs-treated HUVEC cells. (a) Time-dependent normalized capacitance values for HUVECs uptake of different concentrations ($10^8$, $10^9$, and $10^{10}$ PNPs/well) of carboxylate-modified PNPs. The NPs were treated at 24 hours of incubation (gray arrow); the inset graph shows the capacitance peaks after the NPs treatment; the data have been shifted for clarity. The capacitance readings were fitted to the relationship $C \propto f^{-\alpha}$ and $C \propto f^{-\beta}$ in the frequency range of 100 Hz to 1 kHz and 15 kHz to 20 kHz, respectively. b,c, Time-dependent estimates of $|\alpha|$ (b) and $|\beta|$ (c) values from real-time capacitance measurements using our capacitance sensor array. (d) Time-dependent normalized capacitance values, $C/C_o$, where $C_o$ is the initial capacitance for HUVECs pretreated with chlorpromazine and cytochalasin D at 23 hour (violet arrow), 1 hour before the carboxylate-modified PNPs treatment (gray arrow); the inset graph shows the capacitance peaks after the inhibitors and NPs treatments; the data have been shifted for clarity. Time-dependent estimates of $|\alpha|$ (e) and the $|\beta|$ (f) values from real-time capacitance measurements using our capacitance sensor array (n=5).

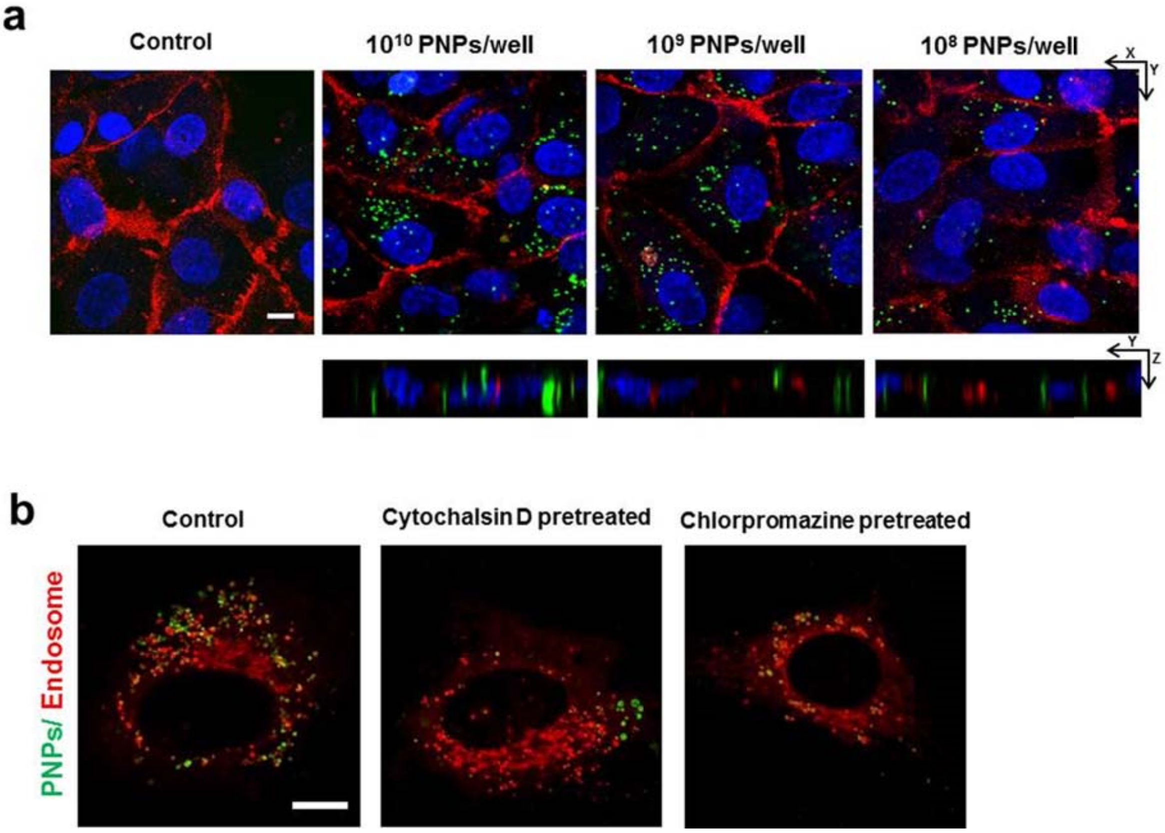


**Supplementary Figure 8.** Images of carboxylate-modified PNPs-treated HUVEC cells. (a) Confocal cross-section and z-stack images of HUVECs showing internalization of $COO^{-}$-PNPs $10^{10}$, $10^{9}$ and $10^{8}$ PNPS/well. Scale bars, 10 μm and stack depth 8 μm. (b) Uptakes of $COO^{-}$-PNPs-FITC (green) by HUVECs and subsequently treated with LysoTracker® Red (left); the same as (b) left, pretreated with cytochalasin D (middle); and the same as (b) left, pretreated with Chlorpromazine (right). The yellow regions indicate co-localization of $COO^{-}$-PNPs with LysoTracker in the superimposed images. Scale bars, 10 μm.

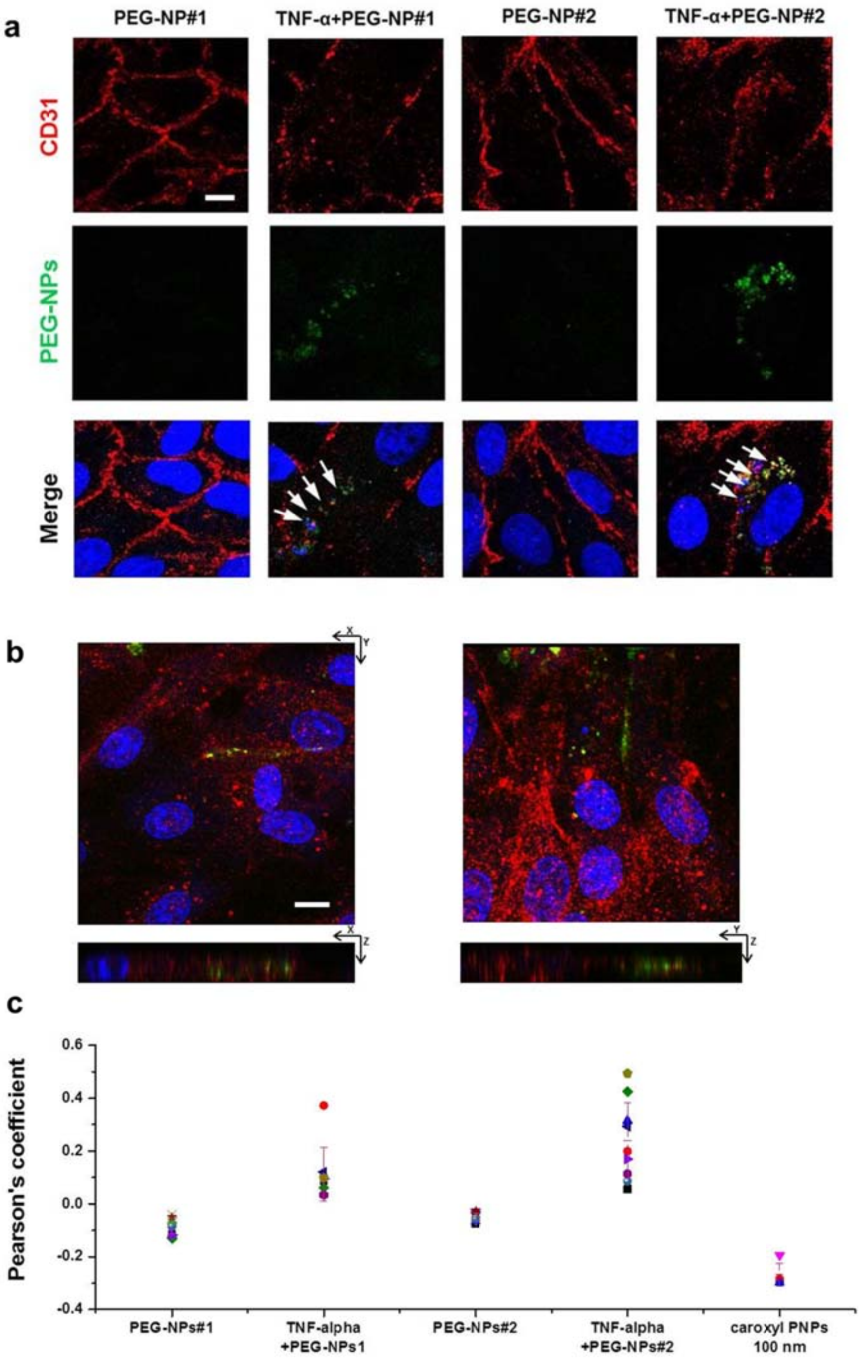


**Supplementary Figure 9.** Images of PEG-NPs-treated HUVEC cells and correlation coefficients. (a) Immunocytochemical staining of a cell membrane with CD31 (red) in HUVEC cells treated with PEG-NPs (green) under different para-cellular permeability states. The white arrows indicate the PEG-NPs alignment with the membrane area. Scale bars, 10 μm. (b) z-stack images of HUVEC cells treated with TNF-α, then with PEG-NPs (green). Scale bars, 10 μm and stack depth, 8 μm. (c) Pearson's correlation coefficient analyses of PEG-NPs#1 and #2 with or without TNF-α, and $COO^{-}$-PNPs (n=9).

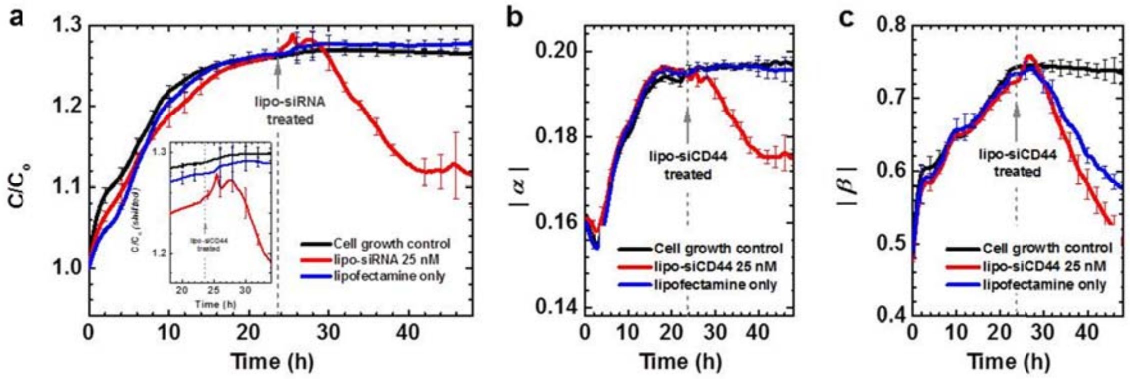


**Supplementary Figure 10.** Time- and frequency-dependent capacitance values in liposome-treated HUVEC cells. (a) Time-dependent normalized capacitance values, $C/C_o$, where $C_o$ is the initial capacitance for HUVECs treated with lipofectamine only and siCD44-lipofectamine at 24 hour (gray arrow). The inset graph shows the capacitance peaks after liposome treatment. After the initial measurement at 100 Hz, capacitance was measured at various frequencies from 100 Hz to 20 kHz. The capacitance readings were fitted to the relationship $C \propto f^{-\alpha}$ and $C \propto f^{-\beta}$ in the frequency range of 100 Hz to 1 kHz and 15 kHz to 20 kHz, respectively. Time-dependent estimates of $|\alpha|$ (b) and $|\beta|$ (c) values from real-time capacitance measurements using our capacitance sensor array (n=5).

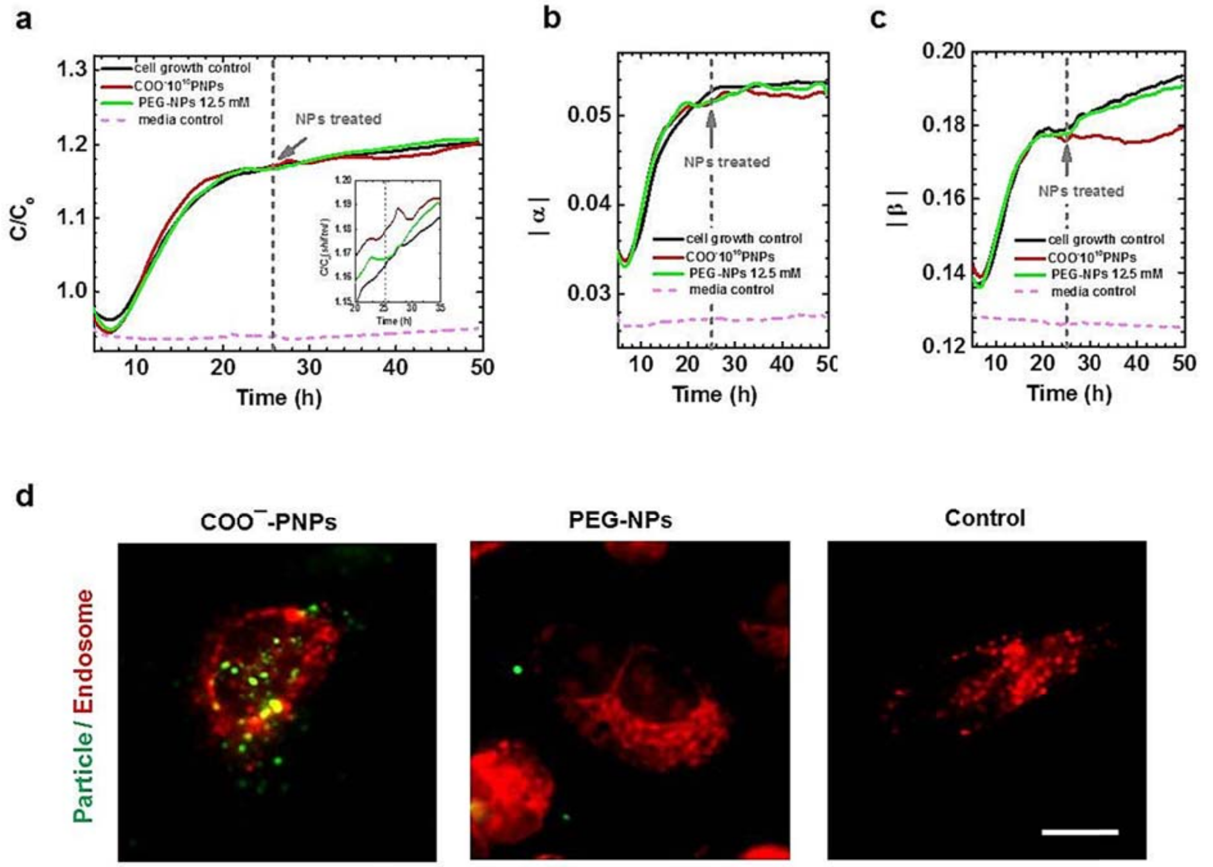


**Supplementary Figure 11.** Time- and frequency-dependent capacitance values in carboxylate-modified PNPs- and PEG-NPs-treated HeLa cells. (a) Time-dependent normalized capacitance values, for uptake of carboxylate-modified PNPs ($10^{10}$ PNPs/well) and PEG-NPs (12.5 mM) by HeLa cells. The NPs were treated at 24 hours of incubation (gray arrow) and the inset graph shows the capacitance peaks after the NPs treatment. The data have been shifted for clarity. Capacitance readings were fitted to the relationship $C \propto f^{-\alpha}$ and $C \propto f^{-\beta}$ in the frequency range of 100 Hz to 1 kHz and 15 kHz to 20 kHz, respectively. Time-dependent estimates of $|\alpha|$ (b) and $|\beta|$ (c) values from real-time capacitance measurements using our capacitance sensor array (n=5). (d) Uptakes of $COO^{-}$-PNPs-FITC (green) by HeLa cells and subsequently treated with LysoTracker® Red (left), PEG-NPs-indocyanine (mid) and no treatment for control (right). The yellow regions indicate co-localization of $COO^{-}$-PNPs with LysoTracker in the superimposed images. Scale bar is 10 μm.

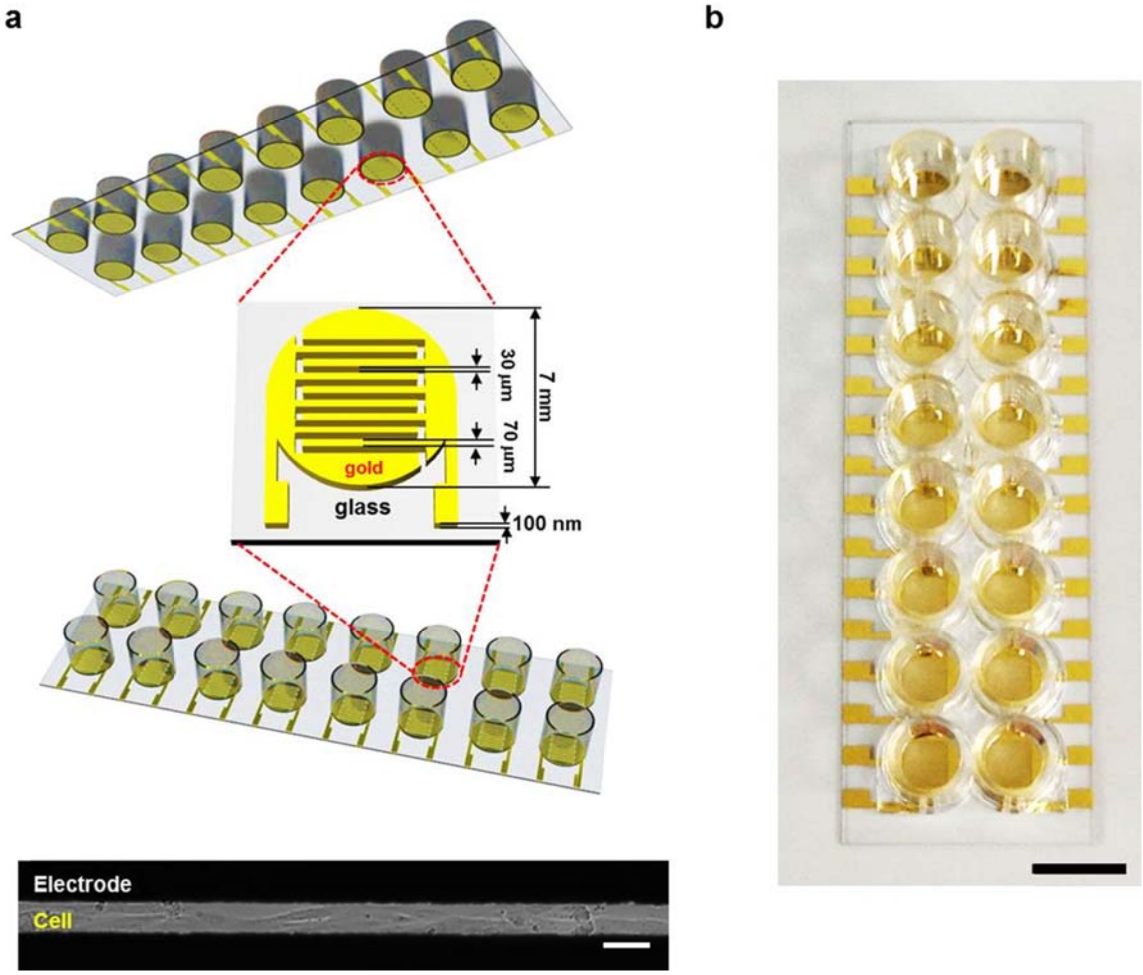


**Supplementary Figure 12.** Schematic diagram of a capacitance sensor array. (a) Schematic diagram of a capacitance sensor array consisting of 16 sensors. The middle diagram shows a capacitance sensor with interdigitated electrodes and an optical image of HUVECs on the interdigitated electrodes. Scale bar is 50 μm. (b) Image of custom made 16-well array sensor. Scale bar is 1 cm.

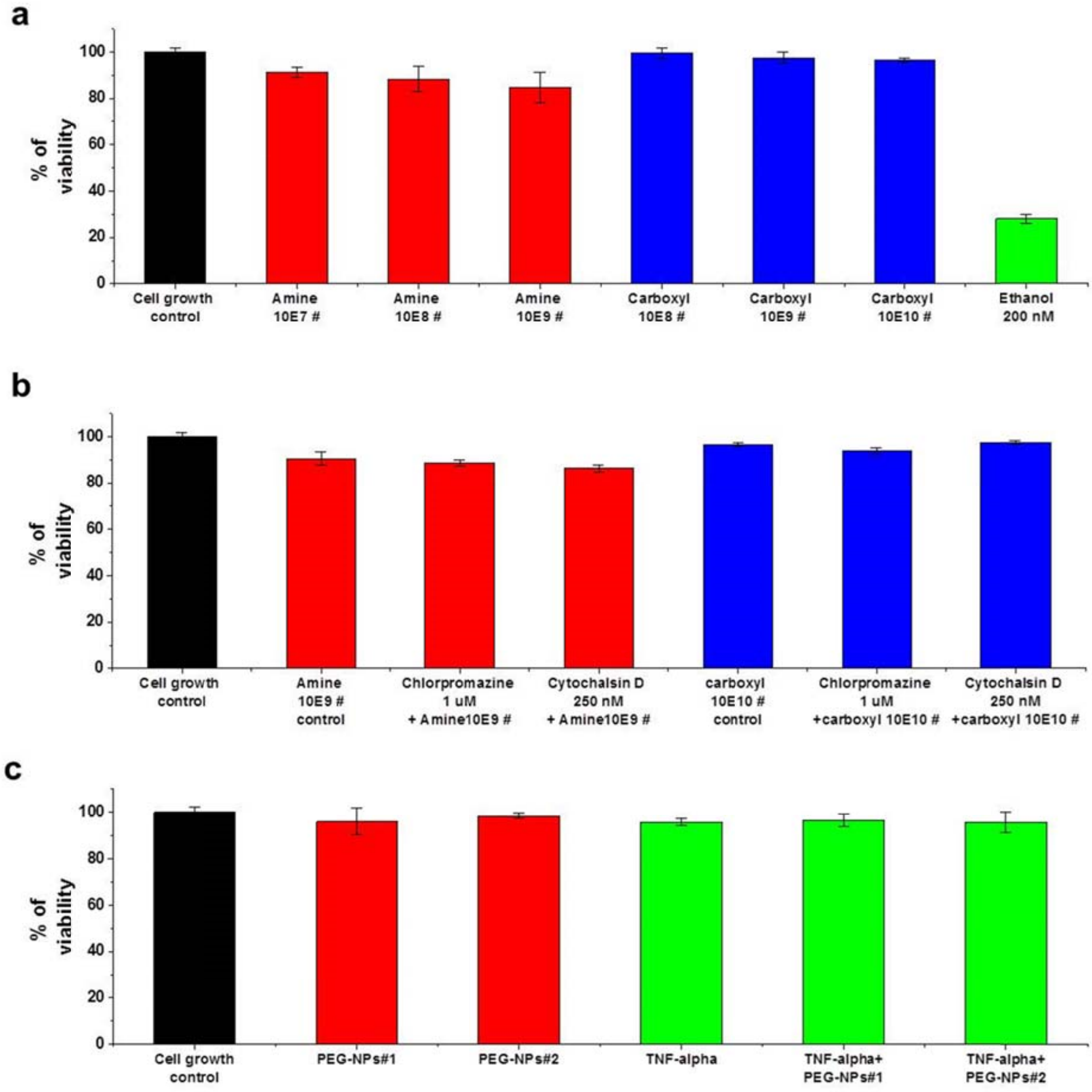


**Supplementary Figure 13.** Cell viability of nanoparticles and chemicals used in this study. Viability measured with the Countess Automated Cell Counter for HUVECs incubated for 24 hours with (a) different concentrations of amine-modified NPs ($10^9$, $10^8$ and $10^7$ PNPs/well) and carboxylate-modified NPs ($10^{10}$, $10^9$ and $10^8$ PNPs/well) (n=5), (b) inhibitor pre-incubated HUVEC cells before amine-modified NPs ($10^9$ PNPs/well) and carboxylate-modified NPs ($10^{10}$ PNPs/well) treatment (n=5) and (c) PEG-NPs#1 and PEG-NPs#2 (12.5 mM) incubated with or without TNF-α (2 ng/mL) (n=5).

**Supplementary Note**

Frequency-dependent capacitance values in cell growth control groups and amine-modified PNPs-treated HUVEC cells with fitting simulations.

When the AC electric field was applied at a low frequency, the cell membrane effectively insulated the cytoplasm, and the electric ion current flowed only around the cell. This was due to the ion current mostly passing through the extracellular spaces. The electrical properties, then, were largely affected by the extracellular volume fraction (Supplementary Fig. 4a). Therefore, at low frequencies, the ion current flow was unaffected by whether the cells uptake the NPs or not (Supplementary Fig. 4b). At high frequencies, however, the cell membrane was more conductive with negligible impedance, so the ion current penetrated the cell membrane and flowed within the intracellular spaces (Supplementary Fig. 4c)[1]. Here, the changes in the composition/volume of the cell cytoplasm and nucleus affected impedance[2]. Thus, the differences in capacitance values at the high-frequency region between the cellular growth control group and cells that uptake NPs could be distinguished because the content of each cell was different. Compared with the cellular growth control group, the cells with NPs uptake had a higher resistive particle and vesicle content, which influenced the ion current flow within the intracellular space (Supplementary Fig. 4d).

To prove this, we measured capacitance as a function of frequency ($f$) while the HUVECs were growing, or after treatment with polystyrene nanoparticles (PNPs). To induce cellular uptake, we seeded 50,000 HUVECs per well; then $10^9$ particles of amine-modified PNPs (positively charged, 200 nm, Supplementary Fig. 1; $NH_3^+$-PNPs) were added to each well. (The $NH_3^+$-PNPs are NPs whose uptake pathways are known to be via endocytic[3].) The

relationship $C \propto f^{-a}$ was observed for all cellular states, with two different exponents at low frequencies ($a$=$\alpha$; 0.1–1 kHz) and high frequencies ($a$=$\beta$; 15–20 kHz) ($C$: capacitance and $f$: frequency). In the log-log plot, $-a$ ($-\alpha$ or $-\beta$) value indicates the slope in the relationship $\log C \propto -a\log f$. As the cells grew (before the NPs treatment), the capacitance was fitted to the relationship $C \propto f^{-\alpha}$ with $|\alpha|\approx0.167$ at t=24 hours and $|\alpha|\approx0.175$ at t=48 hours, with a difference of +0.008 (Supplementary Fig. 3b). As the frequency increased, the capacitance was fitted to the relationship $C \propto f^{-\beta}$ with $|\beta|\approx0.566$ at t=24 hours and $|\beta|\approx0.640$ at t=48 hours, with a difference of +0.08 (Supplementary Fig. 3c).

In contrast, with cellular uptake of NPs, the capacitance conformed to the relationship $C \propto f^{-a}$ with $|\alpha|\approx0.173$ at t=24 hours (immediately before NP treatment) and $|\alpha|\approx0.183$ at t=48 hours, the difference being +0.013 at low frequencies (Supplementary Fig. 3b); and at high frequencies with $|\beta|\approx0.567$ at t=24 hours and $|\beta|\approx0.468$ at t=48 hours, the difference being −0.099 (Supplementary Fig. 3c). A comparison made at 24 and 48 hours for the cellular growth control group showed both the difference in the $|\alpha|$ and $|\beta|$ values to be positive; however, before and after cellular uptake of the NPs, the difference in the $|\alpha|$ values was positive but negative for the $|\beta|$ values. In other words, at a high frequency, the capacitance decreased at a slower rate after cellular uptake of the NPs, as opposed to the cellular growth control group. This is because the cell expresses extremely high dielectric permittivity at low frequencies but gradually decreases at higher frequencies. To better understand the decreased $|\beta|$ values in the high frequency region, we fitted our data using a theoretical electric circuit[4] (Supplementary Fig. 3a); the fitted values are represented by the symbols in Supplementary Figs. 3b and c.

The fitted capacitance values (symbols) fit relatively well to the experimental data (curved line) for the cellular growth control group (Set 1; cell growth at 24 and 48 hours) and the NPs-treated group (Set 2; cell growth at 24 and 24 hours after internalization of $10^9$ particles). The fitted values related to the cell are intracellular bulk resistance ($R_{cyto}$), cell membrane capacitance ($C_m$) and reactive charge transfer resistance on the cell membrane ($R_m$), which are colored red in the electric circuit in Supplementary Fig. 3a. The parameters related to the electrode are reactive charge transfer resistance on the electrode surface ($R_{dl}$); resistance developed on the electrode due to polarization ($R_e$); diffuse double layer capacitance across the electrodes ($C_{dl}$); and constant phase element due to polarization on the electrode surface ($CPE_e$: $CPE_e$-T and $CPE_e$-P). We ignored the resistance and capacitance of the solution because we assumed that the cell and electrode were in very close proximity. In addition, we also ignored membrane polarization because it was very small compared to the resistance and capacitance of the total cell membrane.

In practice, $CPE_e$-P should be in the range of 0 to 1. If it is equal to 1, then $CPE_e$ would be identical to the capacitor[5, 6]. In our study, our fitted values, $CPE_e$-P, obtained from the Set 1 and Set 2 experiments were 0.8978 and 0.8936, respectively. Thus, the $CPE_e$ values obtained in this work are close to the capacitor values, although they are less than 1 due to polarization on the electrode surface ($C_e$, capacitance of electrode surface). Since the experiment electrodes for Set 1 and 2 were different, we obtained different fitted values from the data at 24 hours for each set. However, for each set, the parameter values related to the electrode ($R_{dl}$, $C_{dl}$, $R_e$, $CPE_e$-T and $CPE_e$-P) were fixed to determine the changes to intracellular bulk resistance ($R_{cyto}$), cell membrane capacitance ($C_m$) and reactive charge transfer resistance on the cell membrane ($R_m$) at 48 hours.

For the cellular growth control group without NPs treatment, $R_{cyto}$ decreased by 9.6%. In contrast, once the NPs were internalized, $R_{cyto}$ increased by 25.5%, as the internalized NPs reduced the volume of the ion solution inside the cytoplasm and thereby could be considered electric resistant elements. In other words, the NPs' internalization into the cells increased cytoplasm resistance. $R_m$ and $C_m$ value changes for cellular growth without NPs treatment were -18.6% and -9.2% but after the NPs uptake these changes were -54.6% and -24.8%, respectively. Thus, the NPs' internalization also changed the cellular charge transfer resistance and membrane capacitance. Based on these results, we concluded that changes in $R_{cyto}$, $R_m$ and $C_m$ caused the decreasing capacitance to slow as frequency increased, and led to the decreasing |β| values in the high frequency region. The calculated fitted values are summarized in Supplementary Table 2a and b.

To validate our fitting procedures, we re-calculated the fitted values of the simulated cell growth control group at 48 hours after fixing the parameter values associated with the electrode ($R_{dl}$, $C_{dl}$, $R_e$, $CPE_e$-T and $CPE_e$-P) to the values obtained from Set 2 at 24 hours. Only $R_{cyto}$, $R_m$ and $C_m$ were adjusted according to the percentages (9.6%, -18.6% and -9.2%, respectively) of those obtained from Set 1 at 48 hours (Supplementary Fig. 5b). The |β| values at the high frequency region increased from 0.567 to 0.614 (a change of +8.3%) when $R_{cyto}$, $R_m$ and $C_m$ were adjusted in the cell growth control group, similar to the previously fitted values (a change of +13% in Supplementary Table 2a). The theoretical simulation parameter values are summarized in Supplementary Table 2b: Cell growth/Simulation.

As we described above, the ion current can only flow around the cell at low frequencies. Furthermore, according to the dielectric model, the dielectric constant at low frequencies depends on $v$, $a$, and $\xi$, where $v$ is the volume fraction occupied by the cells, $a$ is the cell

radius and $\xi$ is the zeta potential of the membrane[7]. The cellular activities induce changes in $v$, $a$, and $\xi$, which are affected by changes in the cell membrane area. The changes in this area in turn affect the dielectric constant[8]; thus, the various cellular behaviors associated with the membrane area can be monitored in real time by measuring the change in capacitance at low frequency. To test this, we disturbed only the cell-cell or cell-matrix interactions and simulated the $|\alpha|$ and $|\beta|$ value trends. When we simulated the disturbances in the cell-cell or cell-matrix interactions, we assumed that the changes in intracellular cytoplasm resistance, cell membrane resistance and electrode resistance were small, even negligible. Only the capacitance related parameters ($C_m$, $C_{dl}$ and $CPE_e$) were used for the simulation parameters. In theory, when the cell-cell or cell-matrix interaction decreases, $C_m$ should also decrease. In addition, since a weaker interaction causes increased exposure of the electrode to the ionic solution, $C_{dl}$ and $CPE_e$ should decrease. We simulated changes in the cell-cell or cell-matrix interactions when we fixed the parameter values associated with resistance ($R_{cyto}$, $R_m$, $R_{dl}$ and $R_e$) to the values obtained from Set 1 at 24 hours. Only $C_m$, $C_{dl}$ and $CPE_e$ were adjusted (Supplementary Fig. 6). The $|\alpha|$ values at the low frequency region decreased from 0.167 to 0.146 (a change of −12.6%) when $C_m$, $C_{dl}$ and $CPE_e$ were adjusted to -31%, -92.5% and -12.6 %, respectively, but the $|\beta|$ values increased from 0.566 to 0.604 (a change of +6.71%). The theoretically simulated parameter values are summarized in Supplementary Table 2a: Cell-cell or cell-matrix interactions changes/Simulation. This observation is similar to our experimental results; endothelial cell permeability increased due to the TNF-α treatment and the cell surface adhesion weakened due to the siRNA-mediated depletion of CD44 (Figs. 4b and 5b).

In conclusion, cell permeability or adhesion changes can be observed by measuring the

decrease of the |α| values at the low frequency region, and cellular uptake of NPs can be distinguished by measuring the decrease of the |β| values at the high frequency region.

**Supplementary Table 1.** Difference in $|\alpha|$ and $|\beta|$ values at the low and high frequency regions, respectively, at 24 h and 48 h (NP treatment at 24 h).

| | $\|\alpha\|$ | | $\|\alpha\|$ *difference* | $\|\beta\|$ | | $\|\beta\|$ *difference* |
|---|---|---|---|---|---|---|
| | 24h | 48 h | | 24h | 48 h | |
| Cell growth control | 0.174 | 0.182 | 0.008 | 0.610 | 0.710 | 0.100 |
| Cell growth control+media | 0.175 | 0.187 | 0.012 | 0.646 | 0.719 | 0.073 |
| $NH_3^+$ $10^9$ ps/well treat | 0.167 | 0.168 | 0.001 | 0.579 | 0.557 | -0.022 |
| $NH_3^+$ $10^8$ ps/well treat | 0.165 | 0.167 | 0.002 | 0.579 | 0.565 | -0.014 |
| $NH_3^+$ $10^7$ ps/well treat | 0.164 | 0.166 | 0.002 | 0.594 | 0.590 | -0.004 |
| Chlorpromazine pretreat + $NH_3^+$ ps/well treat | 0.164 | 0.165 | 0.001 | 0.578 | 0.584 | 0.005 |
| Cytochalasin D pretreat + $NH_3^+$ ps/well treat | 0.163 | 0.165 | 0.002 | 0.588 | 0.593 | 0.005 |
| $COO^-$ $10^{10}$ ps/well treat | 0.170 | 0.182 | 0.012 | 0.597 | 0.498 | -0.099 |
| $COO^-$ $10^9$ ps/well treat | 0.168 | 0.181 | 0.013 | 0.597 | 0.566 | -0.031 |
| $COO^-$ $10^8$ ps/well treat | 0.169 | 0.186 | 0.017 | 0.605 | 0.676 | -0.029 |
| Chlorpromazine pretreat + $COO^-$ $10^{10}$ ps/well treat | 0.177 | 0.179 | 0.002 | 0.613 | 0.538 | -0.075 |
| Cytochalasin D pretreat + $COO^-$ $10^{10}$ ps/well treat | 0.178 | 0.183 | 0.005 | 0.633 | 0.621 | -0.012 |

**Supplementary Table 2.** Estimated parameters for capacitance-based cellular sensors and percentage of change for each parameter.

| **a** | Set 1) Cell growth control | | | Cell-cell or cell-matrix interactions changes | |
|---|---|---|---|---|---|
| | Experimental fitting | | | Simulation | |
| | 24 h | 48 h | Change (%) | Interaction change (48 h) | Change (%) |
| $R_{cyto}$ (Ω) | 102.9 | 112.8 | +9.6 | 102.9 | - |
| $R_m$ (Ω) | 18329 | 14914 | -18.6 | 18329 | - |
| $C_m$ (F) | $2.07 \times 10^{-7}$ | $1.88 \times 10^{-7}$ | -9.2 | $1.43 \times 10^{-7}$ | -31 |
| $R_{dl}$ (Ω) | 281.8 | 281.8 | - | 281.8 | - |
| $C_{dl}$ (F)- | $2.95 \times 10^{-8}$ | $2.95 \times 10^{-8}$ | - | $2.2 \times 10^{-9}$ | -92.5 |
| $R_e$ (Ω) | 264.8 | 264.8 | - | 264.8 | - |
| CPEe-T (F) | $4.54 \times 10^{-7}$ | $4.54 \times 10^{-7}$ | - | $2.1 \times 10^{-7}$ | -54 |
| CPEe-P ($n_t$) | 0.8978 | 0.8978 | - | 0.8978 | - |
| $\|\alpha\|$ | 0.167 | 0.175 | +4.7 | 0.146 | -12.6 |
| $\|\beta\|$ | 0.566 | 0.640 | +13 | 0.604 | +6.71 |
| **b** | Set 2) Nanoparticle treatment at 24 h | | | Cell growth | |
| | Experimental fitting | | | Simulation | |
| | 24 h | 48 h | Change (%) | Growth (48 h) | Change (%) |
| $R_{cyto}$ (Ω) | 64.43 | 80.85 | +25.5 | 70.62 | +9.6 |
| $R_m$ (Ω) | 18055 | 8193 | -54.6 | 14696 | -18.6 |
| $C_m$ (F) | $3.83 \times 10^{-7}$ | $2.88 \times 10^{-7}$ | -24.8 | 3.48 × 10-7 | -9.2 |
| $R_{dl}$ (Ω) | 225.5 | 225.5 | - | 225.5 | - |
| $C_{dl}$ (F)- | $9.06 \times 10^{-7}$ | $9.06 \times 10^{-7}$ | - | $9.06 \times 10^{-7}$ | - |
| $R_e$ (Ω) | 262.5 | 262.5 | - | 262.5 | - |
| $CPE_e$-T (F) | $4.61 \times 10^{-8}$ | $4.61 \times 10^{-8}$ | - | $4.61 \times 10^{-8}$ | - |
| $CPE_e$-P ($n_t$) | 0.8936 | 0.8936 | - | 0.8936 | - |
| $\|\alpha\|$ | 0.173 | 0.183 | +5.8 | 0.180 | +4.1 |
| $\|\beta\|$ | 0.567 | 0.458 | -19.2 | 0.614 | +8.3 |

$R_{cyto}$: intracellular (cytoplasm) bulk resistance
$R_m$: reactive charge transfer resistance on the cell membrane
$R_{dl}$: reactive charge transfer resistance on the electrode surfaces
$R_e$: resistance developed on the electrode due to polarization
$C_m$: capacitance across the cell membrane
$C_{dl}$: diffuse double layer capacitance across the electrodes
$CPE_e$-T: admittance magnitude of Constant Phase Element ($CPE_e$).
$CPE_e$-P: exponential term of Constant Phase Element ($CPE_e$).

**REFERENCE**


1. Cone, C.D. The role of the surface electrical transmembrane potential in normal and malignent mitogenesis. *Ann. N. Y. Acad. Sci.* 1974, 238, 420-435.
2. Cheung, K., S. Gawad, and P. Renaud, Impedance spectroscopy flow cytometry: On-chip label-free cell differentiation. *Cytometry A,* 2005, 65A, 124-132.
3. Fazlollahi, F., et al., Polystyrene nanoparticle trafficking across MDCK-II. *Nanomedicine,* 2011,7, 588-594.
4. Amin, M., P.P. Dey, and H. Badkoobehi, A complete electrical equivalent circuit model for biological cell, in Proceedings of the 7th WSEAS International Conference on Applied Computer and Applied Computational Science. 2008, *World Scientific and Engineering Academy and Society (WSEAS)*, 343-348.
5. Chen, A. and S. Nigro, Influence of a Nanoscale Gold Thin Layer on Ti/SnO2-Sb2O5 Electrodes. *J. Phys. Chem. B,* 2003, 10, 13341-13348.
6. Mohammadpour, R., et al., Electrochemically Assisted Photocatalytic Oxidation of Methanol on TiO2 Nanotube Arrays. *J. Mater. Sci. Technol.* 2010, 26, 535-541.
7. Lee, R., et al., Capacitance-based assay for real-time monitoring of endocytosis and cell viability. *Lab Chip.* 2012,12, 2377-2384.
8. Pethig, R., Dielectric Properties of Biological Materials: Biophysical and Medical Applications. *IEEE Trans. Elect. Insulation, 1984*, EI-19, 453-474.
